# Whole genome transcription profiling of *Anaplasma phagocytophilum *in human and tick host cells by tiling array analysis

**DOI:** 10.1186/1471-2164-9-364

**Published:** 2008-07-31

**Authors:** Curtis M Nelson, Michael J Herron, Roderick F Felsheim, Brian R Schloeder, Suzanne M Grindle, Adela Oliva Chavez, Timothy J Kurtti, Ulrike G Munderloh

**Affiliations:** 1 University of Minnesota, Department of Entomology, Saint Paul, Minnesota 55108, USA

## Abstract

**Background:**

*Anaplasma phagocytophilum *(*Ap*) is an obligate intracellular bacterium and the agent of human granulocytic anaplasmosis, an emerging tick-borne disease. *Ap *alternately infects ticks and mammals and a variety of cell types within each. Understanding the biology behind such versatile cellular parasitism may be derived through the use of tiling microarrays to establish high resolution, genome-wide transcription profiles of the organism as it infects cell lines representative of its life cycle (tick; ISE6) and pathogenesis (human; HL-60 and HMEC-1).

**Results:**

Detailed, host cell specific transcriptional behavior was revealed. There was extensive differential *Ap *gene transcription between the tick (ISE6) and the human (HL-60 and HMEC-1) cell lines, with far fewer differentially transcribed genes between the human cell lines, and all disproportionately represented by membrane or surface proteins. There were *Ap *genes exclusively transcribed in each cell line, apparent human- and tick-specific operons and paralogs, and anti-sense transcripts that suggest novel expression regulation processes. Seven *virB2 *paralogs (of the bacterial type IV secretion system) showed human or tick cell dependent transcription. Previously unrecognized genes and coding sequences were identified, as were the expressed *p44/msp2 *(major surface proteins) paralogs (of 114 total), through elevated signal produced to the unique hypervariable region of each – 2/114 in HL-60, 3/114 in HMEC-1, and none in ISE6.

**Conclusion:**

Using these methods, whole genome transcription profiles can likely be generated for *Ap*, as well as other obligate intracellular organisms, in any host cells and for all stages of the cell infection process. Visual representation of comprehensive transcription data alongside an annotated map of the genome renders complex transcription into discernable patterns.

## Background

Arthropod-borne intracellular organisms that parasitize the cells of mammalian hosts must be able to manipulate a diversity of host cells to support their own growth and life cycle. Revealing how they accomplish this will illuminate not only pathogenesis but also cell biology.*Anaplasma phagocytophilum *(*Ap*) is a gram-negative obligate intracellular bacterium, the agent of human granulocytic anaplasmosis (HGA), an emerging tick-borne disease. *Ap *has a 1.47 million base pair genome with 1411 annotated features [1]. Clinically, membrane bound *Ap *colonies, called morulae, are seen in peripheral blood neutrophils. The white-footed mouse (*Peromyscus leucopus*) is considered to be the primary reservoir for the *Ap *variant responsible for HGA, but other mammals are also susceptible [1-4]. Ticks do not pass *Ap *to their offspring, but to mammals they feed upon, which transmit it back to ticks, and so the organism cycles between tick and mammalian hosts.

HGA is a potentially severe illness with symptoms, including pancytopenia and limb edema, that suggest other cells or tissues, beside neutrophils, are infected [5-7] In mice, *Ap *infects endothelial cells [8] and human bone marrow cells support infection *in vivo *and *in vitro *[5,9]. The specific cells infected in ticks have not been unambiguously identified, however evidence indicates they reside within midgut and salivary gland tissues [10-12]. Tick cell lines have been developed that support *Ap *replication, including ISE6, which was isolated from *Ixodes scapularis*, the primary vector of HGA in North America [13]. Susceptible human cell lines include HL-60, a promyelocytic leukemia cell line that serves as a model for neutrophils, and the microvascular endothelial cell line HMEC-1 [14]. *Ap *produces distinct infection phenotypes and growth kinetics in these cell lines, suggesting, along with its broad host range, that the organism adapts to each host by shifting its gene expression.

The obligate intracellular lifestyle of *Ap *makes direct biochemical, genetic, and observational study approaches inherently difficult. Transformation of *Ap *with fluorescent reporters has recently been achieved and should improve visualization of live bacteria, and open avenues for directed genetic research [15]. Nevertheless, methods for functional genomic analysis, for example, specific gene knockout, are still lacking. Gene transcription and expression analyses in animal models are largely impractical because *Ap *levels in tick and mammal tissues are too low for recovery of sufficient bacterial RNA or protein. In vitro studies have focused on characterization of the immunodominant *p44/msp2 *genes, which encode a large family of major surface proteins whose expression varies according to whether the organisms were derived from tick or mammalian host cells [16]. In addition, genes encoding the type IV secretion system of *Ap *have been identified, transcriptionally analyzed, and described [17,18], but their function and regulation remain undefined. DNA microarrays have been used to measure changes in host cell gene transcription during infection, with an aim to infer the mechanisms and strategies applied by *Ap *[19-24], but no microarray studies that directly measure *Ap *transcription have been published.

The release of an annotated *Ap *genome sequence [1], and development of maskless, photolithographic, digital light processor technology (DLP) [25] have made it feasible to characterize global transcript levels in *Ap *using tiling microarrays [26,27]. With these technologies entire genomes can be probed instead of sampling only selected sequences. The continuous data generated can be plotted in genomic order as a line graph, with transcribed genes appearing as peaks rising from a baseline of non-transcribed or intergenic sequence, and peak height corresponding to relative transcript abundance. A direct alignment of this to a parallel, annotated map of the genome can provide a visually striking and intuitive way to assess the data. Through Affymetrix (Santa Clara, CA) and NimbleGen Systems, Inc. (Madison, WI), we designed a tiling microarray for the entire genome of *Ap *(1.47 Mbp) and characterized *Ap *gene transcription in three cell lines representative of its life cycle (ISE6 tick) and pathogenesis in humans (HL-60 and HMEC-1).

## Methods

### Cell lines, *Ap *strain, and growth conditions

Sterile and *Ap*-infected HL-60 cells (American Type Culture Collection, Manassas, VA, USA; ATCC CCL-240) were maintained in RPMI 1640 medium supplemented with 10% fetal bovine serum (FBS) and 25 mM HEPES. Cultures infected with *Ap *isolate HZ were subcultured weekly by 1:50 (v/v) dilution of > 90% infected cells into sterile HL-60 cultures [28]. The HMEC-1 cell line was received from the Centers for Disease Control (Atlanta, GA), and both sterile and infected cells likewise cultured in RPMI 1640 medium with 10% FBS and 25 mM HEPES [29]. Infected HMEC-1 cultures were fed daily and *Ap *subcultured 1:50 bi-weekly when > 80% of cells were infected. HL-60 and HMEC-1 cultures were kept at 37°C in a humidified atmosphere of 5% CO_2 _in air. ISE6 cells were propagated in L15B300 medium with 5% tryptose phosphate broth (BD, Sparks MD, USA), 5% FBS, and 0.1% lipoprotein concentrate (MPBiomedical, Irvine CA, USA) at 34°C [13]. *Ap*-infected ISE6 cultures were fed twice weekly with medium buffered to pH 7.6 using 0.25% NaHCO_3 _and 25 mM HEPES, and subcultured 1:50 bi-weekly [13].

*Ap *strain HZ was cultured from the blood of a New York state patient by co-culture with HL-60 [Goodman et al. unpublished; [28] ] HZ-*Ap*-infected HL-60 cells (passage 8) were simultaneously inoculated into the three cell lines. These infected parallel cultures were continuously subcultured and served as the source of infected cell samples for tiling array analysis. All samples from each cell line were from *Ap *cultures between passages 21 and 34.

### Tiling array design and manufacture

Through consultation with Affymetrix (Santa Clara, CA), a library of 258,480 complimentary (perfect match) 25-mer oligonucleotide probes covering both DNA strands of the *Ap *genome (isolate HZ) [1] was designed. Each probe overlapped its neighbor by 11 bases for a probe resolution of 14 bases, the distance from the center of one probe to the next. Probes were "hard pruned" – ridden of highly repetitive sequence elements thought to be irrelevant using an algorithm (Affymetrix) to identify, somewhat subjectively, long repeat sequences. Probes for these were not included, though probes for many "shorter" repeating sequences were. Pruned sequences can be viewed easily in the Artemis graphs. They are characterized by successive data points with the same or similar value that together produce large blunt peaks. For examples see additional file 1 coordinates 665858–666184, 1025792–1026289, and 645698–646032. NimbleGen Systems, Inc. (Madison, WI) synthesized the oligonucleotide probes *in situ *using a photo-mediated, maskless process in which the synthesis of each probe is directed by a digital light processor [25].

### Isolation of RNA

**Figure 1:**
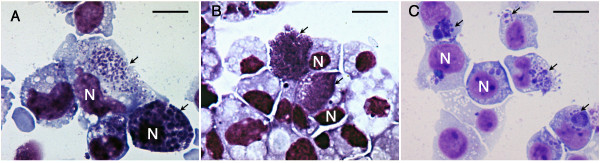
Microscopic images of Giemsa stained cells infected with *Ap*. (A) *Ap*-infected HMEC-1 (B) *Ap*-infected ISE6 (C) *Ap-*infected HL-60. Cell nuclei are labeled "N" and arrows point to *Ap *morulae. Scale bar = 10 μm

*Ap *genomic transcription was measured in each of the three cell lines when cultures were approximately 95% infected. Typically, cells contained hundreds of bacteria (Figure 1: Microscopic images of Giemsa stained cells infected with *Ap*). RNA was extracted from three *Ap*-infected and three uninfected samples of each cell line (18 samples total). Each sample was from a separate culture and consisted of approximately 10^7 ^infected cells or uninfected control cells. Cells were suspended by pipetting (HL-60 and ISE6) or with a cell scraper (HMEC-1) and immediately centrifuged at 300 × g for 2 minutes. The supernatant was aspirated and discarded; cell pellets were loosened by flicking and immediately dissolved in TRI REAGENT™ (Sigma, Saint Louis, MO, USA). All steps were performed at room temperature. Total RNA was then isolated according to the TRI REAGENT™ product instructions. In brief, samples in TRI REAGENT™ were extracted with chloroform and centrifuged at 12,000 × g for 15 minutes at 4°C. RNA in the aqueous, upper phase was precipitated in isopropanol, collected by centrifugation at 12,000 × g for 10 minutes at 4°C, and washed twice in cold 75% ethanol. RNA pellets were dissolved in 100 μL RNase-free water, quantified by spectrophotometry, and processed for array analysis.

### Preparation of tiling array "target"

Total RNA from *Ap*-infected or sterile control cells was processed for *Ap *transcript measurement according to the Affymetrix "Prokaryotic Target Preparation" protocol using random priming of total RNA to synthesize a single strand of cDNA. The cDNA was recovered by column purification, fragmented with DNase I, and end labeled with biotin. These biotinylated cDNA fragment "targets" were hybridized to the "probes" contained on the tiling arrays, labeled with a streptavidin-phycoerythrin conjugate, and probe hybridization was quantified by laser scanning. The detailed protocol was as follows.

### cDNA synthesis

In a volume of 30 μL, 10 μg of total RNA (from *Ap *infected or sterile control cells) was combined with random primers (25 ng/μL final concentration) (Invitrogen, Carlsbad, CA), and, in a thermocycler, incubated 10 minutes at 70°C followed by 10 minutes at 25°C, then chilled to 4°C. To this reaction mixture was added 30 μL of the following master mix: 12 μL 5× 1^st ^Strand Buffer, 6 μL 100 mM DTT, 3 μL 10 mM dNTPs, 1.5 μL SUPERaseIn™ (20U/μL) (Ambion, Austin TX, USA), 7.5 μL SuperScript II (200 U/μL) (Invitrogen). Samples (60 μL) were incubated in a thermocycler 10 minutes at 25°C, 60 minutes at 37°C, 60 minutes at 42°C, 10 minutes at 70°C, and chilled to 4°C.

### cDNA isolation and fragmentation

To degrade RNA, 20 μL of 1N NaOH was added to each sample, incubated at 65°C for 30 minutes, and neutralized by addition of 20 μL 1N HCl. MiniElute PCR Purification Columns (Qiagen, Valencia CA, USA) were used according to product instructions to purify cDNA from the samples. Typical cDNA yields were 3–4 μg. cDNA in 10 μL was combined with 2 μL 10× One-Phor-All Buffer (Amersham Biosciences, Piscataway, NJ), 0.6 U DNase I/μg cDNA (Amersham Biosciences), plus sufficient water for 20 μL total volume, and incubated 10 minutes at 37°C. DNase I was inactivated by heating to 98°C for 10 minutes. cDNA fragments produced were 50–200 bases in length.

### Biotinylation of 3' termini of cDNA fragments

The GeneChip^® ^DNA labeling kit (Affymetrix) was used as follows: 20 μL fragmented cDNA was combined with 10 μL 5× reaction buffer, 2 μL 7.5 mM GeneChip DNA labeling reagent, 2 μL terminal deoxynucleotidyl transferase, and 16 μL water and incubated at 37°C for 60 minutes. The reaction was stopped with 2 μL of 0.5 M EDTA and then frozen at -20°C until it was applied to an array.

### Tiling array hybridization and scanning

Samples were hybridized to tiling arrays and scanned at the BioMedical Genomics Center at the University of Minnesota using the Affymetrix Fluidics Station 400. Arrays were scanned using an Affymetrix Genechip 3000 scanner according to standard Affymetrix protocols.

### Tiling array data analysis

"Cel" files generated by the University of Minnesota's microarray facility were joined to Affymetrix BPMAP files specific to the tiling array using Affymetrix^® ^Tiling Analysis Software (TAS). TAS generated a list of signal intensities and arranged them in order of genomic location and DNA strand. The data are available at the NCBI Gene Expression Omnibus (GEO) database (study #GSE11487 ).

Graphical representation of these data along with their annotations was accomplished with the JAVA based program "Artemis" . Using a script developed internally, the intensity plots were reformatted and imported into Artemis along with an annotation feature list . The resulting graphics give a visual overview of transcription as it relates to genomic organization, and provide clues to operon structure (see additional file 1: Artemis transcription graph of the entire, annotated *Ap *genome during infection of HL-60, HMEC-1, and ISE6 cell lines). The complete genome coverage provided by the overlapping probes on the tiling array translates into 90 spot intensities generated for a 1000 base open reading frame (ORF). This large number of intensities, coupled with the quality of data suggested that creating a linear graph, and measuring the area under the peaks in regions corresponding to annotated open reading frames – ORF transcription areas – would be a simple and useful method to quantify transcripts for each ORF. To compute these ORF transcription areas, the intensities were normalized via quantiles [30] and imported into the IgorPro data analysis program (WaveMetrics Lake Oswego OR, USA) along with the ORF and structural RNA annotations available from . A script was written to index a trapezoidal integration algorithm of the intensity list with the start and end genomic positions indicated on the annotation. This script operation generated a list of 1411 transcription areas.

### Statistical evaluation of area differences, T values & Fold change

ORF transcription areas computed from the quantile normalized data (3 each for HL-60, HMEC-1 and ISE6) and paired 2 tail Students t-test, were performed on: HL-60 vs. ISE6, HMEC-1 vs. ISE6, and HL-60 vs. HMEC-1. ORF transcription area comparisons with p values ≤ 0.05 were considered significant for determination of the number and identity of genes transcribed. Determination of differentially expressed genes utilized the additional requirement that the mean ORF transcription area be at least twice the mean ORF transcription area of the same gene of the compared cell line.

The number of expressed ORFs was determined by T-test comparison between the ORF transcription areas from infected cell monolayers, and those of uninfected control cell monolayers. The signal intensity of these arrays was baseline corrected using the signal intensities of twelve manually selected intergenic regions devoid of obvious signal from across the span of the genome. ORF transcription area comparisons with p values ≤ 0.05 were considered significant for determination of the number and identity of genes transcribed.

### Validation of tiling array data by quantitative reverse transcription-PCR (qRT-PCR)

**Figure 2:**
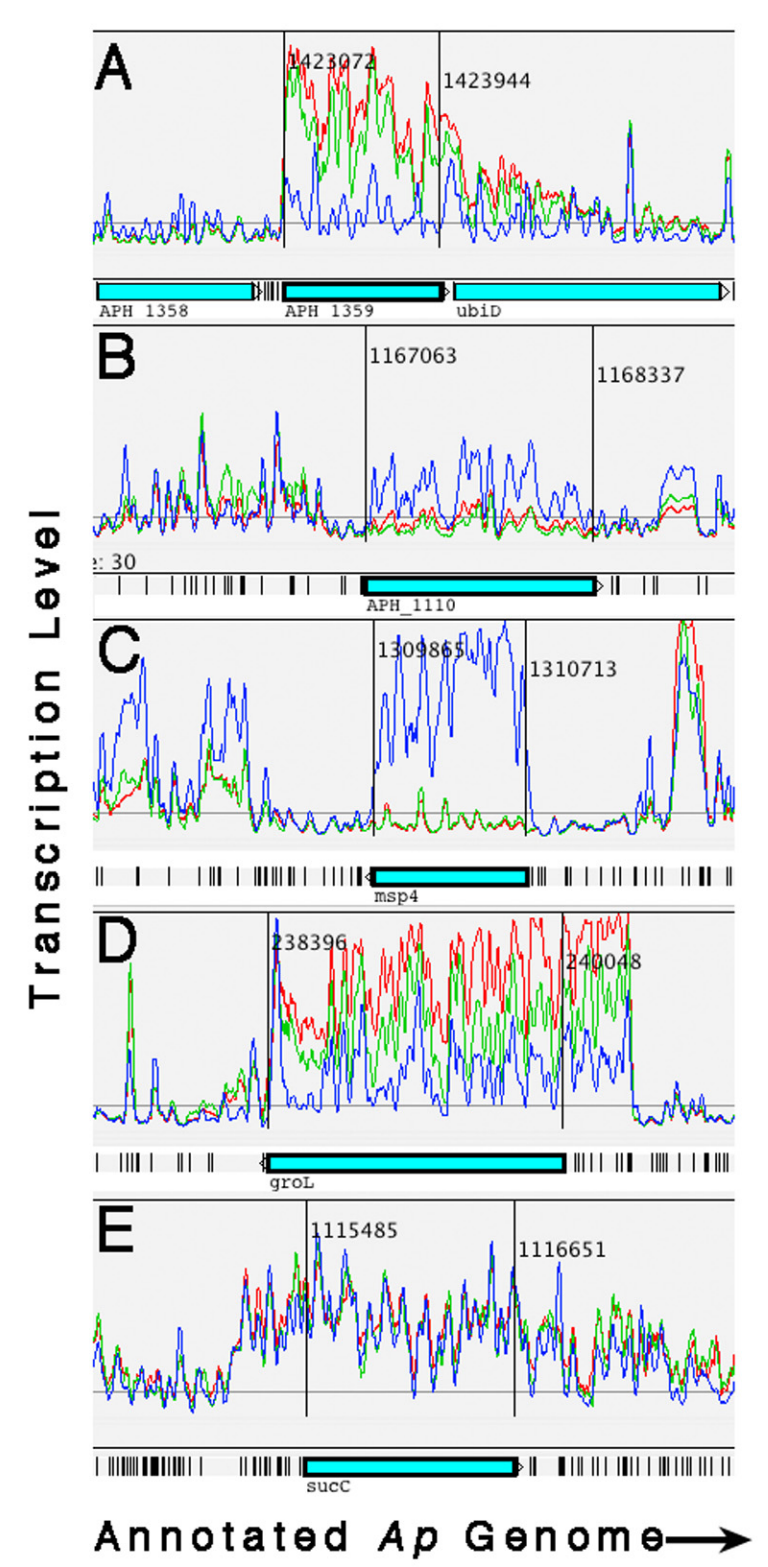
Artemis profiles depicting the relative transcription levels of five *Ap *genes during infection of HL-60 (red), HMEC-1 (green), and ISE6 (blue) cells. Plots were "smoothed" by setting the sliding window average to 5. (A) Major outer membrane protein gene (*omp-1A*; *APH_1359*) transcription greater in the human cell lines compared to the tick cell line. (B) Outer membrane efflux protein (*APH_1110*) greater in the tick cell line compared to the human cell lines. (C) Transcription of the major surface protein 4 gene (*msp4*; *APH_1240*) only in the tick cell line. (D) Transcription of the 60 kDa chaperonin gene (*groL*; *APH_0240*) was greatest in HL-60, significantly lower in HMEC-1, and least in ISE6. (E) Equal transcription of the succinyl-CoA synthetase beta subunit gene (*sucC*; *APH_1052*) in all three cell lines.

Five *Ap *genes with known products were assayed for relative transcript abundance by qRT-PCR. Tiling data indicated that four of the genes had differential transcription patterns between the human and tick cells: major outer membrane protein (*omp-1A*; *APH_1359*), outer membrane efflux protein (*APH_1110*), major surface protein 4 (*msp4*; *APH_1240*), and the 60 kDa chaperonin (*APH_0240*). The fifth gene, which codes for succinyl-CoA synthetase beta subunit (*APH_1052*), was transcribed equally in all three cell lines (Figure 2; Artemis transcription profiles for five genes chosen for assay by qRT-PCR).

Total RNA (from portions of samples prepared for array analysis), from three separate cultures of each *Ap*-infected cell line (9 samples), were assayed in triplicate by qRT-PCR. To eliminate any DNA contamination, samples were DNase I treated using DNA-free™ (Ambion). DNase I was inactivated and RNA purified using RNeasy mini columns (Qiagen, Valentia, CA). mRNA from each of the five genes was reverse transcribed and amplified quantitatively with primers designed using MacVector (Cary, NC) and Netprimer (Palo Alto, CA) (see additional file 2: qRT-PCR primers). The primers were tested by conventional PCR on a Stratagene (La Jolla CA, USA) Robocycler with temperature gradient capability, using *Ap *strain HZ DNA as target. Formation of appropriate product sizes was verified and a single annealing temperature (60°C) and primer concentration (150 nM) suitable for all five primer pairs were determined, allowing RNA from each of the cell lines to be qRT-PCR-amplified together for best determination of relative transcript levels. Reverse transcription and subsequent quantitative PCR were performed on 100 ng of each RNA sample in 96-well plates using the Brilliant II SYBR Green 1-step qRT-PCR kit (Stratagene), and Stratagene's Mx3005P thermal cycler. To initiate the qRT-PCR, reverse transcription was allowed to proceed for 30 minutes at 50°C, followed by heat treatment for 10 minutes at 95°C to activate DNA polymerase and deactivate reverse transcriptase. cDNA was then amplified during 40 cycles of 30 seconds at 95°C, 1 minute at 60°C, and 1 minute at 72°C.

## Results

### Percentage of *Ap *genes measured as transcribed in each cell line

Of the 1411 annotated features [1] in the *Ap *genome, 983 (69.6%) were significantly transcribed (p-value ≤ 0.05) in HL-60, 620 (43.9%) in HMEC-1, and 974 (69.0%) in ISE6, compared to negative control samples (RNA from uninfected cells).

### Differential *Ap *gene transcription between cell lines

Between HL-60 and HMEC-1, 71 *Ap *ORFs (5%) were differentially (p-value ≤ 0.05) transcribed (see additional file 3: *Ap-*HL-60 vs. *Ap*-HMEC-1 differential transcription). Between HL-60 and ISE6, 585 *Ap *ORFs (41.5%) were differentially transcribed. Between HMEC-1 and ISE6, 304 *Ap *ORFs (21.5%) were differentially transcribed. Adding a fold change criterion of 2 or greater, only one *Ap *gene between *Ap *from HL-60 (*Ap*-HL-60) and *Ap *from HMEC-1 (*Ap*-HMEC-1) passed: *APH_1342*, one of the *p44*/*msp2 *paralogs. Between *Ap*-HL-60 and *Ap *from ISE6 (*Ap*-ISE6), 117 ORFs (8.5%), and between *Ap*-HMEC-1 and *Ap*-ISE6, 61 (4.3%) were at least 2-fold different (Table 1). The relatively low percentage of ORFs measured as transcribed in *Ap*-HMEC-1 (43.9%) was probably due to lower average signal intensity from those samples (850 vs. 2120 in HL-60). We determined this to be the result of suboptimal biotin labeling after using a particular batch of terminal transferase. A new aliquot of terminal transferase used in the preparation of one of the samples of *Ap*-HL-60 produced a particularly bright signal, resulting in a higher signal to noise ratio for the *Ap-*HL-60 data. Because of this, and because differential transcription was low between the human cell lines (compared to that between the human and tick cell lines), subsequent descriptions of differential transcription in *Ap*-ISE6 are based on comparisons to *Ap-*HL-60.

**Table 1:** Summary of differential *Ap *gene transcription between HL-60, HMEC-1, and ISE6

	# ORFs Differentially transcribed (p ≤ 0.05)	% of total ORFs	# ORFs ≥ 2-fold (p ≤ 0.05) differentially transcribed	% of total ORFs
*Ap*-HL-60 vs. *Ap*-HMEC-1	**71**	5.0	**1**	0.07
*Ap*-HL-60 vs. *Ap*-ISE6	**585**	41.5	**117**	8.5
*Ap*-HMEC-1 vs. *Ap*-ISE6	**304**	21.5	**61**	4.3

Summary of differential *Ap *gene transcription between HL-60, HMEC-1, and ISE6.

Of the 117 *Ap *ORFs differentially transcribed (p ≤ 0.05, ≥ two-fold difference) between the HL-60 and ISE6 cells, 76 had higher levels in HL-60 and 41 had higher levels in ISE6. The 76 *Ap*-HL-60 ORFs comprise 35 known and 41 hypothetical proteins (54%). All but three of the ORFs that were up-regulated in *Ap*-ISE6 are annotated as hypothetical (93%) (see Table 2: Genes differentially transcribed between human (HL-60) and tick (ISE6) cells). By comparison, 40% of all *Ap *genes are annotated as hypothetical.

**Table 2:** Summary of *Ap*-HL-60 vs. *Ap*-ISE6 differential gene transcription

	**Gene Product**	**Locus**	**Predicted Cellular Location**	**Fold Change Ap-HL-60/Ap-ISE6**
1	DNA-binding protein	APH_1100	Cytoplasmic	4.9
2	HGE-14 protein	APH_0387	**Extracellular**	4.8
3	hypothetical protein	APH_1412	**Outer Membrane**	4.2
4	hypothetical protein	APH_0915	**Outer Membrane, Extracellular**	4.1
5	hypothetical protein	APH_0906	**Outer Membrane**	4.1
6	major outer membrane protein OMP-1A	APH_1359	**Outer Membrane**	4.0
7	hypothetical protein	APH_1378	**Outer Membrane**	3.8
8	hypothetical protein	APH_0842	Cytoplasmic	3.7
9	hypothetical protein	APH_0838	**Outer Membrane**	3.6
10	hypothetical protein	APH_0388	Cytoplasmic	3.6
11	hypothetical protein	APH_1145	**Inner Membrane**	3.5
12	OmpA family protein	APH_0338	**Outer Membrane**	3.4
13	DNA-binding response regulator	APH_1099	Cytoplasmic	3.4
14	hypothetical protein	APH_1144	**Inner Membrane**	3.4
15	hypothetical protein	APH_0837	Cytoplasmic	3.4
16	HGE-14 protein	APH_0382	**Extracellular**	3.3
17	hypothetical protein	APH_0005	**Inner Membrane**	3.3
18	hypothetical protein	APH_0756	**Inner Membrane, Cytoplasmic**	3.2
19	10 kDa chaperonin	APH_0241	**Periplasmic**	3.2
20	hypothetical protein	APH_0032	**Outer Membrane, Extracellular**	3.1
21	hypothetical protein	APH_0874	**Outer Membrane**	3.1
22	hypothetical protein	APH_0233	**Inner Membrane**	3.1
23	HGE-14 protein	APH_0385	Cytoplasmic	3.1
24	signal peptidase II	APH_1160	**Inner Membrane**	3.0
25	hypothetical protein	APH_1156	Cytoplasmic	3.0
26	hypothetical protein	APH_0793	**Inner Membrane**	2.9
27	HGE-14 protein	APH_0455	**Extracellular**	2.9
28	Omp-1N	APH_1220	**Outer Membrane**	2.8
29	hypothetical protein	APH_0949	**Inner Membrane, Cytoplasmic**	2.7
30	hypothetical protein	APH_0033	Cytoplasmic	2.7
31	hypothetical protein	APH_1307	**Inner Membrane**	2.7
32	hypothetical protein	APH_1157	**Inner Membrane**	2.7
33	hypothetical protein	APH_1151	**Inner Membrane**	2.6
34	antioxidant AhpC/Tsa family	APH_0795	Cytoplasmic	2.6
35	RNA polymerase sigma-32 factor	APH_0759	Cytoplasmic	2.6
36	60 kDa chaperonin	APH_0240	Cytoplasmic	2.6
37	hypothetical protein	APH_1235	Cytoplasmic	2.5
38	hypothetical protein	APH_0922	**Inner Membrane**	2.5
39	hypothetical protein	APH_1262	Cytoplasmic	2.5
40	hypothetical protein	APH_0757	Cytoplasmic	2.5
41	chaperone protein DnaK	APH_0346	Cytoplasmic	2.4
42	hypothetical protein	APH_1236	Cytoplasmic	2.4
43	hypothetical protein	APH_0363	Cytoplasmic	2.4
44	translation initiation factor IF-3	APH_1263	Cytoplasmic	2.4
45	glyceraldehyde-3-phosphate dehydrogenase type I	APH_1349	Cytoplasmic	2.4
46	hypothetical protein	APH_0873	Cytoplasmic	2.4
47	hypothetical protein	APH_0919	**Inner Membrane**	2.4
48	hypothetical protein	APH_1072	Cytoplasmic	2.4
49	hypothetical protein	APH_1320	Cytoplasmic	2.3
50	HGE-14 protein	APH_0453	Cytoplasmic	2.3
51	outer membrane protein MSP2 family	APH_1325	**Outer Membrane**	2.2
52	hypothetical protein	APH_0643	Cytoplasmic	2.2
53	hypothetical protein	APH_0839	**Outer Membrane**	2.2
54	putative acyl carrier protein	APH_0929	Cytoplasmic	2.2
55	Es1 family protein	APH_0006	Cytoplasmic	2.2
56	hypothetical protein	APH_0179	Cytoplasmic	2.2
57	iron-sulfur cluster assembly accessory protein	APH_0676	Cytoplasmic	2.2
58	putative ATP synthase F0 B' subunit	APH_1190	Cytoplasmic	2.1
59	hypothetical protein	APH_0719	Cytoplasmic	2.1
60	hypothetical protein	APH_0991	Cytoplasmic	2.1
61	succinate dehydrogenase cytochrome b556 subunit	APH_0999	**Inner Membrane**	2.1
62	pyruvate phosphate dikinase	APH_0185	Cytoplasmic	2.1
63	iron-binding protein	APH_0051	Cytoplasmic	2.1
64	nucleoside diphosphate kinase	APH_1217	Cytoplasmic	2.1
65	malonyl CoA-acyl carrier protein transacylase	APH_0092	Cytoplasmic	2.0
66	hypothetical protein	APH_0786	Cytoplasmic	2.0
67	co-chaperone GrpE	APH_0036	Cytoplasmic	2.0
68	hypothetical protein	APH_0771	Cytoplasmic	2.0
69	hypothetical protein	APH_0585	Cytoplasmic	2.0
70	hypothetical protein	APH_0655	Cytoplasmic	2.0
71	ribonucleoside-diphosphate reductase alpha subunit	APH_0331	Cytoplasmic	2.0
72	P44-45 outer membrane protein	APH_0171	**Outer Membrane**	2.0
73	adenylosuccinate lyase	APH_0867	Cytoplasmic	2.0
74	P44-36 outer membrane protein	APH_1168	**Outer Membrane**	2.0
75	aspartate aminotransferase	APH_0660	Cytoplasmic	2.0
76	cytochrome C membrane-bound	APH_0180	**Periplasmic**	2.0
	**35/76 named genes ≥ 2-fold up in *Ap*-HL-60 = 54% hypothetical**		**33/76 genes membrane associated = 43%**	
77	hypothetical protein	APH_0197	**Periplasmic**	0.5
78	hypothetical protein	APH_0369	Cytoplasmic	0.5
79	hypothetical protein	APH_0497	Cytoplasmic	0.5
80	hypothetical protein	APH_0425	Cytoplasmic	0.5
81	hypothetical protein	APH_0587	Cytoplasmic	0.5
82	hypothetical protein	APH_0963	Cytoplasmic	0.5
83	hypothetical protein	APH_1130	**Inner Membrane**	0.5
84	hypothetical protein	APH_0467	Cytoplasmic	0.5
85	thiamine biosynthesis protein ThiC truncation	APH_0586	Cytoplasmic	0.5
86	hypothetical protein	APH_0806	**Periplasmic**	0.5
87	hypothetical protein	APH_0599	Cytoplasmic	0.5
88	hypothetical protein	APH_0827	Cytoplasmic	0.4
89	outer membrane efflux protein	APH_1110	**Outer Membrane**	0.4
90	hypothetical protein	APH_1131	**Inner Membrane**	0.4
91	hypothetical protein	APH_0829	Cytoplasmic	0.4
92	hypothetical protein	APH_0818	Cytoplasmic	0.4
93	hypothetical protein	APH_0841	Cytoplasmic	0.4
94	hypothetical protein	APH_1382	Cytoplasmic	0.4
95	hypothetical protein	APH_0550	Cytoplasmic	0.4
96	hypothetical protein	APH_0485	Cytoplasmic	0.4
97	hypothetical protein	APH_0355	**Inner Membrane**	0.4
98	hypothetical protein	APH_1132	**Inner Membrane**	0.3
99	hypothetical protein	APH_0720	**Outer Membrane**	0.3
100	hypothetical protein	APH_1384	**Outer Membrane**	0.3
101	hypothetical protein	APH_1380	Cytoplasmic	0.3
102	hypothetical protein	APH_1370	Cytoplasmic	0.3
103	hypothetical protein	APH_0320	Cytoplasmic	0.3
104	hypothetical protein	APH_0726	**Membrane**	0.3
105	hypothetical protein	APH_1369	Cytoplasmic	0.3
106	hypothetical protein	APH_1368	Cytoplasmic	0.3
107	hypothetical protein	APH_1385	Cytoplasmic	0.2
108	hypothetical protein	APH_0724	**Membrane**	0.2
109	hypothetical protein	APH_0805	**Outer Membrane**	0.2
110	hypothetical protein	APH_0723	**Membrane**	0.2
111	hypothetical protein	APH_0487	**Inner Membrane**	0.2
112	hypothetical protein	APH_1386	Cytoplasmic	0.2
113	hypothetical protein	APH_0177	**Extracellular**	0.1
114	hypothetical protein	APH_0546	**Extracellular**	0.1
115	major surface protein 4	APH_1240	**Outer Membrane**	0.1
116	hypothetical protein	APH_0916	**Inner Membrane**	0.1
117	hypothetical protein	APH_0406	**Outer Membrane**	0.1
	**3/41 named genes ≥ 2-fold up in *Ap*-ISE6 = 93% hypothetical**		**19/41 genes membrane associated = 46%**	

Genes differentially transcribed (p ≤ 0.05, ≥ two-fold difference) between human (HL-60) and tick (ISE6) cells. Gene products (117) are listed in descending order of their transcript abundance in HL-60, with their fold change indicated in the right hand column. Gene products 1–76 were those more highly transcribed in HL-60, and 77–117 were those more highly transcribed in ISE6. Gene products predicted to be membrane associated (in bold) tended to be those most differentially transcribed – at the top (most abundant in *Ap*-HL-60) and bottom (most abundant in *Ap*-ISE6) of the list. The percentages of hypothetical genes, and the percentages of gene products that are membrane associated are also indicated. (~25% of all *Ap *genes code for proteins that are membrane associated.)

The amino acid sequences derived from the 117 differentially transcribed genes were analyzed using the secretomeP CBS prediction server [31] and the CELLO subcellular localization predictor [32] to determine the probable cellular location of each of the gene products – periplasm, inner or outer membrane, extracellular (secreted), or cytoplasmic. While 25% of all *Ap *genes products are membrane associated (non cytoplasmic), 43% of the 76 genes differentially transcribed in HL-60 cells and 46% of the 41 genes differentially transcribed in ISE6 cells code for non cytoplasmic proteins. As illustrated in Table 2, the greater a gene's differential transcription, the more likely it was to encode a membrane associated protein (i.e. differentially transcribed genes were over-represented by membrane associated proteins; see Table 2: Summary of *Ap*-HL-60 vs. *Ap*-ISE6 differential gene transcription).

**Figure 3:**
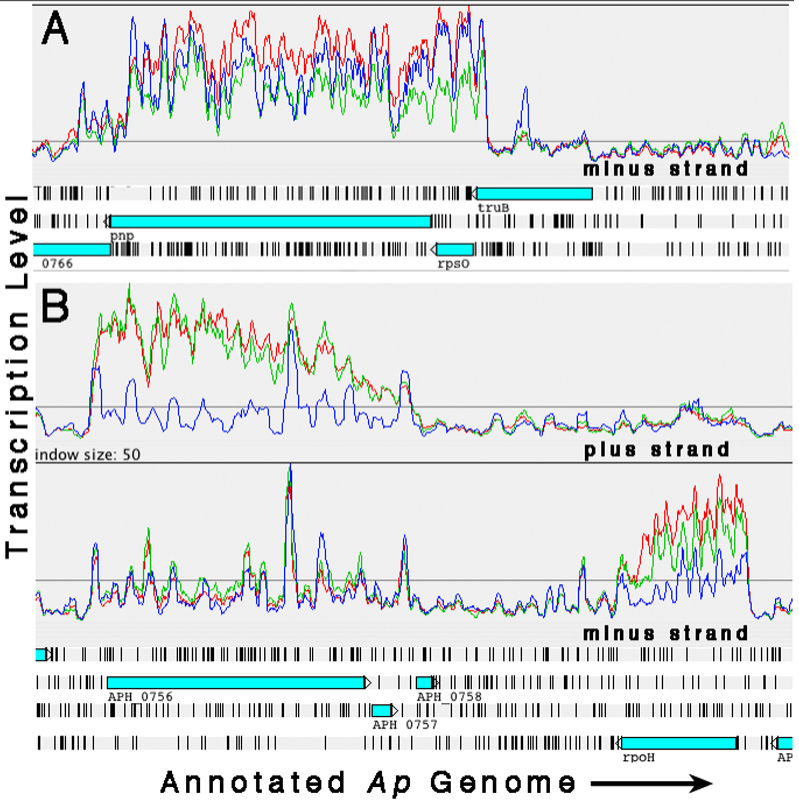
Artemis transcription plots showing examples of flat and sloped gene transcription profiles (Red: *Ap*-HL-60, Green: *Ap*-HMEC-1, Blue: *Ap*-ISE6; plots were "smoothed" by setting the sliding window average to 5). (A) Polynucleotide phosphorylase gene (*pnp*) with an over-all flat transcription profile in all three cell lines. (B) Two examples of genes – *APH_0756 *(hypothetical) and *rpoH *(heat shock sigma factor sigma 32) – with transcription profiles that slope downward from 5' to 3'.

As illustrated in the Artemis transcript level graphs (see additional file 1: materials for graphing transcript level data in Artemis), when the data are displayed as linear graphs alongside a map of the annotated genome, numerous transcription behaviors are revealed. Transcribed sequences are seen to rise from the over-all flat baseline and generally correspond well to annotated ORFs. However, there are examples of transcript signal extending beyond ORF boundaries (*APH_ *numbers *0005, 0406, 0793, 0808, 0811, 0859, 0906*, and *1151*), transcription apparently not associated with an ORF (coordinates 46672–46738, 944100–944549, 692299–692983, and 1306128–1306875), and transcribed unannotated ORFs (875684–876751, 1445252–1445797 and 1241148–1241727). The ORF identified between coordinates 1241148 and 1241727 is another *p44/msp2 *paralog, bringing the total number of *p44 *loci now identified to 114 (113 were originally annotated; [1]. Peaks and plateaus of varying profile representing gene transcription are clearly discernible. Often they slope downward from 5' to 3', but sometimes they are flat (Figure 3: Examples of flat and sloped transcription peaks). There are also numerous ORFs and operons that showed no significant transcription in any of the cell lines (see additional file 4: genes and operons with no detected transcripts).

**Figure 4:**
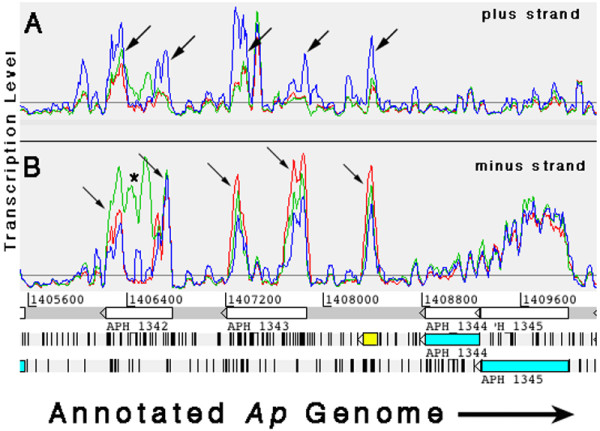
Artemis transcription plots of characteristic *p44 *transcription profiles (Red: *Ap*-HL-60, Green: *Ap*-HMEC-1, Blue: *Ap*-ISE6; plots were "smoothed" by setting the sliding window average to 5). Arrows in panel B indicate *p44 *conserved sequence "horns" on the coding (minus) strand, and "reflected" horns (panel A) in the anti-sense (plus) strand. A strong signal (green) associated with the HVR in *APH_1342 *(*), likely indicates expression of the corresponding *p44 *paralog (*p44-31*) in HMEC-1. The lack of HVR associated signal in *APH_1343*, but strong conserved sequence associated signals (horns), is typical of most *p44 *paralogs. An unannotated segment of *p44 *conserved sequence lies between *APH_1343 *and *APH_1344 *(yellow) on the minus strand. It also showed strong sense (B) and anti-sense (A) signals. *APH_1344 *and *APH_1345 *show typical transcription profiles: signal on the sense strand (B) but not on the anti-sense strand (A).

Paralogs of the *p44/msp2 *family of outer membrane proteins form a characteristic hybridization pattern that is somewhat perplexing. Since *p44 *is abundantly expressed in *Ap*, transcripts with sequences that correspond to the conserved ends of the gene should bind to all the probes on the array that are complimentary – i.e., those of over 100 genes. Signals associated with the conserved ends of the *p44 *paralogs do rise sharply, while those that correspond to the hypervariable region (HVR) in between are generally near baseline. This produces a double horn shaped signature. Most paralogs are not expressed within a population of bacteria [33] therefore those that display bridged horns – representing transcript hybridization to the HVR – are likely to be specifically transcribed. In HL-60, *APH_1152 *(similar to p44-47) and *APH_1351 *(similar to *p44-35*), and in HMEC-1, *APH_1253 *(similar to *p44-39*), *APH_1342 *(similar to *p44-31*), and *APH_1350 *(similar to *p44-51*) had strong signals associated with their HVRs, suggesting those paralogs were expressed. *Ap*-ISE6 produced no significant hybridization to any of the *p44 *HVRs, however along with *Ap*-HL-60 and *Ap-*HMEC-1, *Ap*-ISE6 produced strong signals to the conserved *p44 *sequences. In all three cell lines, signals to the conserved *p44 *sequences were greater than those from the HVRs – of the expressing paralogs noted in *Ap*-HL-60 and *Ap*-HMEC-1. In addition, this pattern of excessive hybridization to the conserved ends of the *p44 *ORFs, is "reflected" in the non-coding DNA strand. Probes to sequences opposite conserved *p44 *sense sequences are hybridized significantly in the human cell samples, and as strongly in the tick cells as the sense probes, such that the horned profile appears reflected in the opposite DNA strand. (Figure 4: *p44 *transcription phenomena: horns, reflecting, and HVR associated signal)

**Figure 5:**
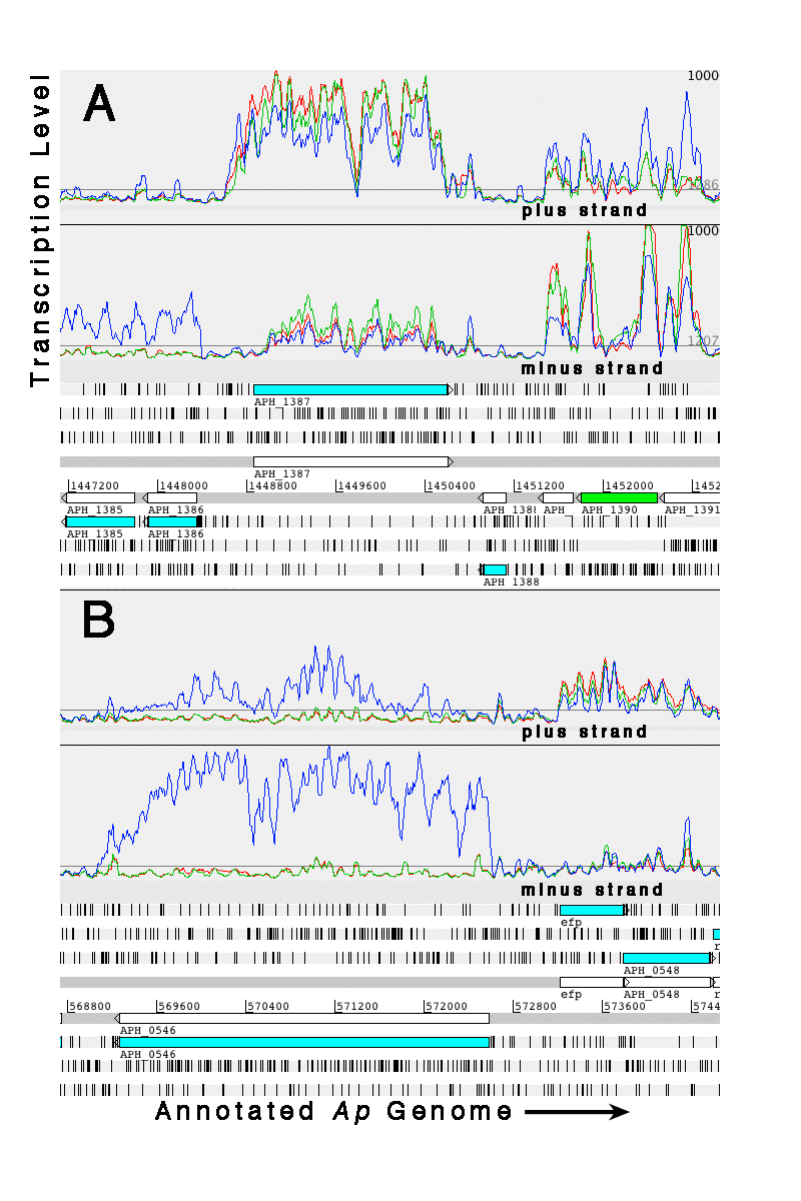
Artemis transcription plots of two genes showing "reflecting" transcription patterns on the anti-sense strands (Red: *Ap*-HL-60, Green: *Ap*-HMEC-1, Blue: *Ap*-ISE6; plots were "smoothed" by setting the sliding window average to 5). (A) HGE2 protein *APH_1387*. (B) Hypothetical protein APH 0546. Note that *Ap *in all three cell lines produced sense and anti-sense transcript for *APH_1387 *(panel A), while in the case of *APH_0546 *(panel B) only *Ap*-ISE6 produced sense and anti-sense transcript.

Exceptions are *p44-70, p44-71, p44-72, and p44-79*, which have "conserved" ends that differ significantly from the other *p44s*; they produced no horns or reflections (see additional file 1, coordinates 680648–684696 and 1418814–1420199). Subtler reflecting was also seen in several non-*p44 *ORFs, such as *APH_1387*, which codes for outer membrane protein HGE2 [1], and the hypothetical *APH_0536 *(Figure 5: Reflecting).

Like conserved *p44 *sequences, repeat sequences, which are common throughout the genome, generally displayed strong signals on both DNA strands (see additional file 5: Repeat-sequence-based sense and anti sense signal).

**Figure 6:**
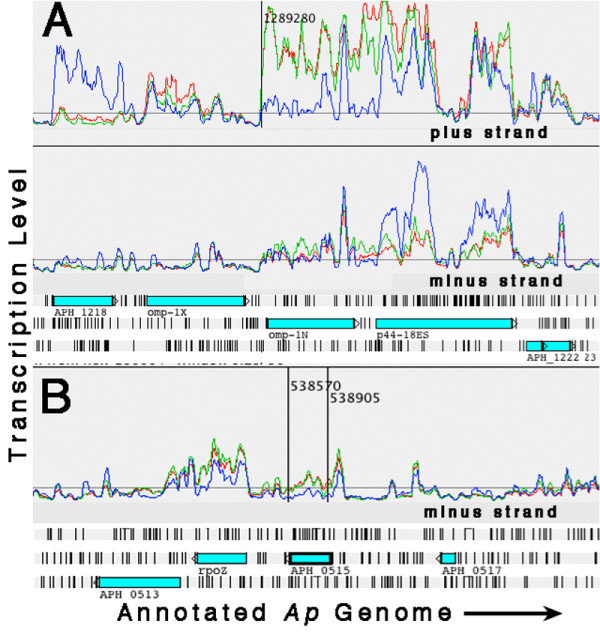
Artemis plots illustrating transcription activity at the *p44 *expression site, and at *ApxR*, a putative *p44 *transcription regulator. (Red: *Ap*-HL-60, Green: *Ap*-HMEC-1, Blue: *Ap*-ISE6; plots were "smoothed" by setting the sliding window average to 5). (A) In the human cell lines, *Ap *shows transcription beginning upstream of *omp-1N *(and *p44-18ES*, the *p44 *expression locus) near coordinate 1289280, but there is no specific transcription in the tick cell line. Transcription regulator *tr1 *(*APH_1218*) is not transcribed in the human cell lines but is in the tick cell line. (B) *ApxR *(*APH_0515*), a putative regulator of *p44 *transcription – through binding to and inhibiting the *tr1 *promoter – shows low-level transcription in the human cell lines but none in the tick cell line.

At the *p44 *expression locus (*APH_1221*) both *Ap*-HL-60 and *Ap-*HMEC-1 showed strong transcription beginning near base 1289280, just before the start of the *omp-1N *gene, and continuing through the *p44 *expression site, while *Ap*-ISE6 did not. The *p44 *"horns" seen in *Ap*-ISE6 within the expression locus, are likely examples of the generalized hybridization to conserved *p44 *sequence noted above. The *tr1 *gene (*APH_1218*) upstream of the *p44 *expression locus, which encodes a putative transcription regulator [34], is well transcribed by *Ap-*ISE6 but not by *Ap*-HL-60 or *Ap*-HMEC-1. The DNA binding protein *ApxR *(*APH_0515*; [34] was weakly transcribed in the human cell lines but not at all in the tick cell line (Figure 6: Artemis transcription plots of the *p44 *expression site, and *ApxR*, a putative *p44 *transcription regulator).

**Figure 7:**
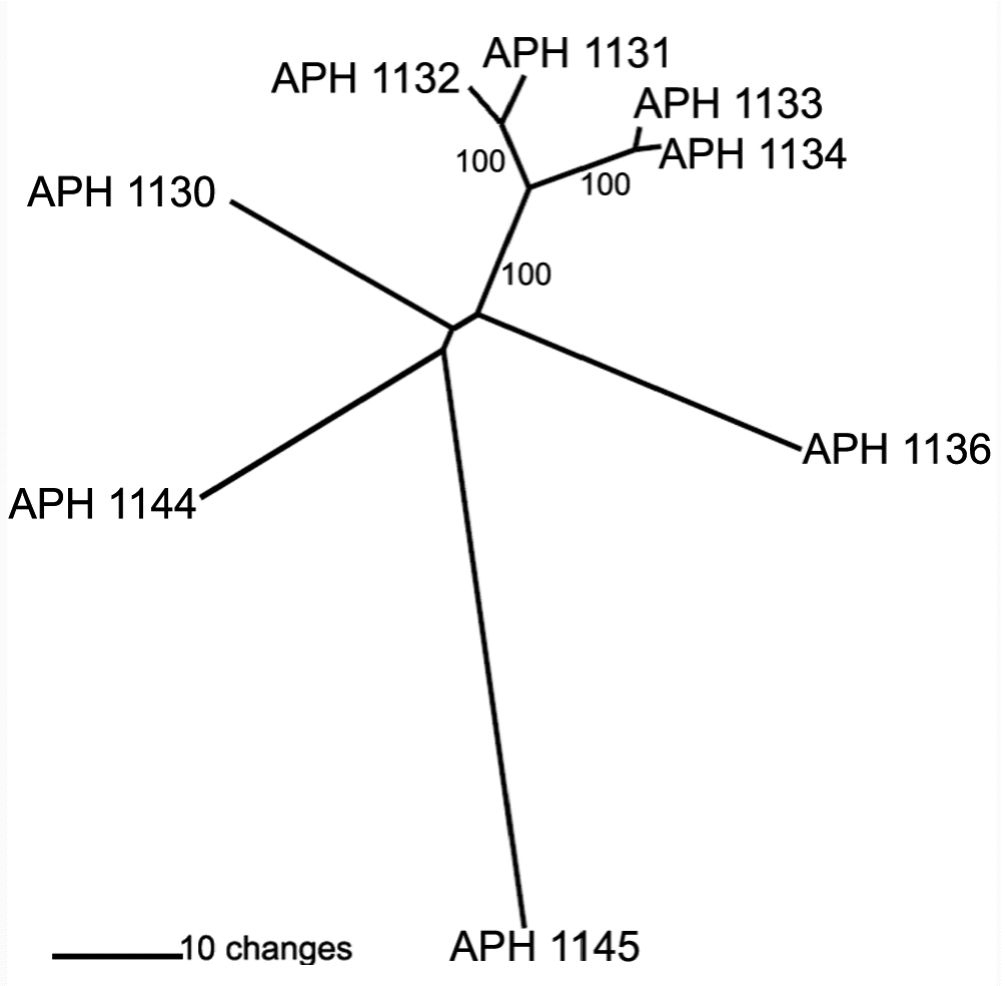
Phylogenetic tree showing the relationship, based on amino acid sequence, of eight *virB2 *paralogs in the *Ap *genome. Four were transcribed only in ISE6 (*APH_1131 – APH_1134*), and two only in HL-60 and HMEC-1 (*APH_1144 *and *APH_1145*). No transcript from *APH_1130 *or *APH_1136 *was measured. The tick cell line associated paralogs are closely related to each other, while those transcribed in the human cell lines form a separate group and are less related to each other. The tree was constructed with PAUP 4.0 using neighbor-joining: absolute variation. Values shown in branches correspond to 2000 bootstraps analysis.

The type IV secretion system genes identified by Hotopp et al. [1] consistently showed little activity in any of the host cells, while *sodB *(*APH_0371*), an iron superoxide dismutase shown to be co-transcribed with components of the type IV secretion system of *E. chaffeensis *and *Ap *[18], was moderately transcribed by *Ap *in all three cell lines. *Ank *(*APH_0740*) was strongly transcribed in *Ap*-HMEC-1, somewhat less so in *Ap-*HL-60, and only marginally in *Ap*-ISE6. This *Ap *gene encodes a protein that is translocated to the nucleus of infected HL-60 cells [35,36] and phosphorylated there within minutes [37], presumably as an effector molecule delivered via the *Ap *type IV secretion system [38]. Located between genome coordinates 1194300 and 1203600 are eight paralogs of the *TrbC/VirB2 *gene family (pfam04956), six of which showed measurable transcript levels either only in the tick cell line (*APH_1131 – APH_1134*), or the human cell lines (*APH_1144 *and *APH_1145*). The relationship by amino acid sequence of these eight paralogs is illustrated in Figure 7 (Phylogenetic tree of eight *virB2 *paralogs by amino acid sequence), and indicates those transcribed in ISE6 are more closely related to each other than those transcribed in HL-60 and HMEC-1. Amino acid sequence alignments for the eight *virB2 *paralogs of *Ap *(see additional file 6) show identities that rank from a high of 93% between tick cell expressed paralogs *APH_1133 *and *APH_1134*, and a low of 22% between non-expressed *APH_1136 *and human cell expressed *APH_1145*. Multiple alignment showed higher identity and similarity between the C termini of paralogs, which contain the functional portion of the proteins.

Two apparent tick-cell-specific operons were identified. ORFs between coordinates 1448342 and 1445170, which include locus tags *APH_1386 *through *APH_1382*, were transcribed only in the tick cell line (see additional file 7: Tick- and human-specific *Ap *operons). Locus tag *APH_1380 *appears to be part of the operon and as such was transcribed in the tick cell line, and, at a lower level in the human cell lines. The functions of the hypothetical proteins of these six ORFs are not known. However, a BLAST homology search produced E values of 9e-18 to 4e-9, indicating the six ORFs are related. The transcription profile around *APH_1380 *and sequence characteristics just up-stream, suggest that the ORF actually begins with the methionine at coordinate 1445107. In support of this, there is a ribosomal binding site at coordinate 1445120. This upstream area shows significant amino acid sequence homology with the N-termini of the other ORF members of this putative operon, also suggesting the sequence is part of that ORF. Between coordinates 1445252 and 1445797 an un-annotated ORF appears to be transcribed only in the tick cell line, and also shows significant homology to the other ORFs in this putative operon. If this is a true ORF, and the start of *APH_1380 *is extended to coordinate 1445107, the two putative ORFs *APH_1381 *and *APH_1382 *on the positive DNA strand may not be true ORFs, since they are situated opposite coding sequences in the operon and showed no transcription signal (see additional file 7 panel A). The other apparent tick specific operon includes locus tags *APH_0726 *through *APH_0720 *(see additional file 7 panel B). All but the small locus tags *APH_0721 *and *APH_0722 *were transcribed. Although these genes are also annotated as encoding hypothetical proteins, searches using SignalP [39] and TMHMM [40] prediction servers indicated they all have transmembrane domains. There was also a group of *Ap *genes transcribed only in the human cells: *APH_0837*, *APH_0838*, *APH_0839*, and *APH_0842 *(see additional file 7 panel C). All encode hypothetical proteins and all are related by amino acid sequence, especially *APH_0838*, *APH_0839*, and *APH_0842*.

### qRT-PCR

**Figure 8:**
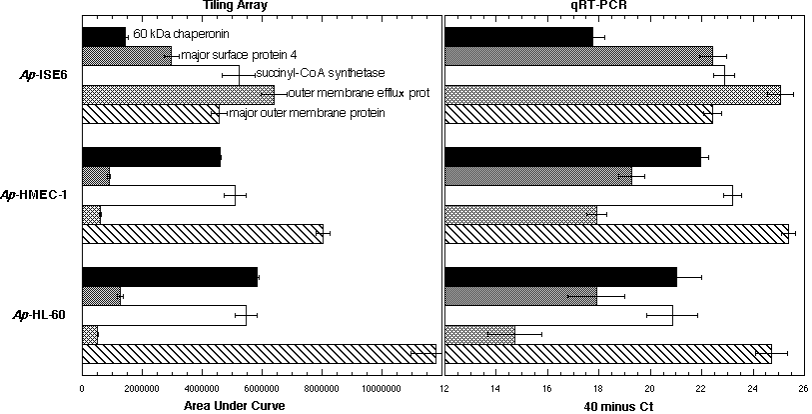
Tiling array (area under gene curve) vs. qRT-PCR (40 minus threshold cycle) measurements of transcript levels of five *Ap *genes (key to bars indicated) during growth in HL-60, HMEC-1, and ISE6 cells. Relative transcript levels for the five selected *Ap *genes, within and between cell lines, confirm those indicated by the array data. qRT-PCR data was converted by subtracting the Ct (threshold cycle) from forty (total PCR cycles), since lower threshold cycles correspond to higher transcript levels.

Relative transcript levels for the five selected *Ap *genes, within and between cell lines, confirm those indicated by the array data (Figure 8; Tiling vs. qRT-PCR graphs).

## Discussion

Total RNA from *Ap *infected human and tick cells was used to establish host cell specific *Ap *transcription profiles by hybridization to complementary oligonucleotides representing the entire genome of *Ap *on tiling arrays. The high percentages of genes measured as transcribed (69.6% in HL-60, 43.9% in HMEC-1, and 69.0% in ISE6), and the low levels of hybridization produced by the uninfected control samples, demonstrate that the method and array design produced sensitive, consistent, and specific transcription measurements. This is encouraging since efforts to fractionate or amplify RNA samples inevitably skew results. However, the culture samples analyzed were heavily infected and therefore optimal for such a direct approach. The three cell lines – HL-60 human promyelocytic, HMEC-1 human microvascular endothelial, and ISE6 tick – each produced bacteria with distinct transcription profiles, suggesting that *Ap *gene expression is closely dependent on the phenotype and genotype (species origin) of its host cell. The bacteria assayed were not synchronized, they were the result of 1:50 inoculations, and therefore the transcription profiles generated were an average, perhaps with a "late stage" bias, of the infection process in each cell line.

Transcription profiles between the two human cell lines appeared similar, however with better and more consistent biotin labeling the percentage of *Ap *ORFs transcribed in HMEC-1 (43.9%) is predicted to be closer to that seen in HL-60 and ISE6 (~70%), and differences in transcription profiles between *Ap*-HL-60 and *Ap*-HMEC-1 would be magnified to reveal additional essential characteristics of *Ap *transcription in the human promyelocytic versus endothelial cells. Transcription differences between the human and tick cells were extensive; there were many genes and apparent operons transcribed in the tick cells but not in the human cells, and vice versa. The fact that the vast majority of tick cell specific transcripts are for hypothetical genes is tantalizing, and likely reflects our ignorance of the molecular patho-physiology of ticks and their associated bacteria.

The observation that in all three cell lines some *Ap *genes and operons remained inactive, is either an indication that there are genetic capabilities not called for by these in vitro infection conditions – the particular intracellular environments of each cell line and the laboratory growth conditions – or the failure of this method to measure the transcription of those genes. Genes and operons that were truly silent may, among other possibilities, encode products specific to earlier stages of infection, to colonization of ticks following blood-meal uptake, or to parasitism of different hosts. Given the distinct transcription profiles produced between the human and tick cells, and the diversity of animal hosts and cell types infected within each, all are possible explanations.

The *virB2 *paralogs of the type IV secretion system (T4SS) identified as differentially transcribed (6 of 8) between the human and tick cells (*APH_1144 *and *APH_1145*, and *APH_1131 – APH_1134*, respectively) represent host cell specific usage of type IV secretion system components. *VirB2 *is the major protein that makes up the T4SS pilus, and has been shown to be necessary for full virulence in *Brucella abortus *[41]. In *Ap*, seven of the eight *virB2 *paralogs are annotated as being *TrbC/VirB2 *(*pfam04956*) family members on the Entrez Protein entries for each individual protein. *APH_1145*, although not annotated as *virB2*, shares homology with and is located next to the other seven. Several other bacteria within the family *Anaplasmataceae *also possess multiple paralogs of *virB2*, which is unusual, as the majority of bacteria with type IV secretion systems have only one or two *virB2 *genes. A blast search done with *APH_1133 *shows, for example, that *Anaplasma marginale*, as well as *Ehrlichia *and *Wolbachia *species, also have multiple loci annotated as *TrbC/VirB2 *family members (see additional file 8: Examples of other *Anaplasmataceae *bacteria with multiple *virB2 *loci). These bacteria might also express specific *virB2 *paralogs in a host cell dependent manner.

The absence of *p44 *transcription in ISE6 at the *p44 *expression locus and clear transcription in HL-60 and HMEC-1, is consistent with the observation that the tick cell samples produced little or no hybridization to *p44 *HVRs, while the human samples did, and indicates that in ISE6 little if any transcript was generated from any of the 22 full-length *p44 *genes. The lack of *ApxR *transcript in the tick cells is consistent with the findings of Wang et al., who performed quantitative reverse transcription PCR on *Ap*-infected ISE6 cells and tick salivary glands and found that *ApxR *is not transcribed [34]. It was suggested that *ApxR *generally regulates transcription in mammalian host cells and specifically regulates *p44 *transcription by binding to the *tr1 *promoter. The strong transcription of *tr1 *in the tick cells in this study may be due to a lack of suppression by *ApxR*, which is not transcribed in the tick cells. The function of *tr1*, therefore, is unclear.

**Figure 9:**
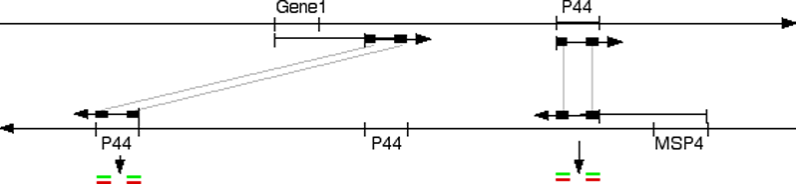
A proposed model for generation of the observed anomalous *p44 *conserved sequence transcripts (sense and anti-sense). "Read through" transcription of genes lying just upstream of anti-sense *p44 *sequence (e.g. "*Gene 1*" and *msp4*) may produce anti-sense *p44 *transcript, which, together with *p44 *sense transcript, forms double stranded RNA (dsRNA). Because the HVR sequences are not complementary they do not form dsRNA and are therefore degraded. However, the conserved, complementary sequences do form dsRNA so are stabilized, accumulate in the bacteria, and are measured as over-abundant by the arrays.

The apparent over-representation of transcript from conserved *p44 *sequences, along with its reflecting behavior in the anti-sense strand, is unexpected. It may be the result of transcriptional "read-through" followed by the formation of stable double stranded, conserved sequence RNA. Bacteria are known to have poor control over transcription termination, and transcription of anti-sense sequence has been identified in *Mycoplasma genitalium *[42]. Since *p44 *paralogs are scattered throughout the genome on both DNA strands, any adjacent gene transcription that continues into sense or anti-sense *p44 *sequences will create "false transcripts," the conserved sequences of which are complementary. Conserved anti-sense false transcript may anneal to conserved sense "true" and false transcript to form double stranded conserved sequence RNA, which is relatively stable compared to single stranded RNA and thus would accumulate in the bacteria (Figure 9: Diagram of possible mechanism to explain the over-representation of *p44 *conserved sequence transcripts and their anti-sense counterparts). Sense and anti-sense *p44 *false transcripts could come from many of the numerous *p44 *paralogs, but a possible source of anti-sense *p44 *transcript in the tick cells is via read-through from the *msp4 *gene (see additional file 9: *msp4 *transcription), which is opposite and just downstream of *p44-15b *and *p44-13*, strongly transcribed in the tick cells, not transcribed in the human cells, and has no obvious transcription terminator.

It is possible that the anti-sense transcription noted in some genes, along with the prominent *p44 *transcription phenomena, function to regulate gene expression. In prokaryotes, *cis*- and *trans*-encoded anti-sense transcripts regulate coding sequence lying directly opposite or elsewhere in the genome, respectively [43]. Although anti-sense mediated expression regulation mechanisms are poorly understood, some possible modes have been discussed and include: imprinting through DNA methylation, RNA processing interference, and ribosome interference [44,45]. In the case of *p44*, anti-sense transcripts may serve to silence leaky expression occurring from any of the 22 identified full-length *p44 *paralogs [1], which are apparently capable of being expressed independently from the *p44 *expression locus [46]. *P44 *silencing may be especially important in tick cells and account for the particular abundance of anomalous *p44 *conserved sequence transcripts in *Ap*-ISE6, which showed no *p44 *HVR transcription. Sense and anti-sense RNA homologous to the conserved ends of the *p44 *genes may even facilitate the process of non-reciprocal recombination by which *p44 *paralogs move into and out of the expression locus [47]. If they are not purposeful, it is likely that these gene transcription phenomena are the result of poorly controlled transcription or are artifacts of the tiling arrays. The repeat sequence associated sense and anti-sense "transcripts" do appear to be an artifact of the tiling arrays, as they are consistently seen wherever repeat sequences occur, whether inside or outside of coding sequences. However, the transcription behavior of *p44 *is unique in the genome, and most genes do not display anti-sense transcription, therefore the over-representation of transcript to conserved *p44 *sequences and its reflection, and the anti-sense transcription of some genes, are intriguing and merit further investigation.

## Conclusion

Obligate intracellular pathogens like *Ap *control the cells they parasitize – to prevent immune attacks, divert cellular resources, and prevent host cells from apoptosing. Our understanding of tick genes is poor so it is not surprising that the up-regulated *Ap *genes in tick cells are nearly all "hypothetical." Matched with our limited understanding of *Ap *genes, the tick cell data are particularly difficult to interpret. Conversely, it makes sense that the most differentially active *Ap *genes in HL-60 cells are better characterized, since human cell lines have mainly been used to study the biology of *Ap*, and, perhaps, *Ap *genes evolved to interact in human cells would tend to be related to characterized effectors. It also makes sense that the differentially transcribed *Ap *genes in HL-60 and ISE6 are over-represented by membrane associated gene products, since survival in such disparate host cells would seem to require substantial specialization at the interface of the organism with its host cell: the bacterial membrane. The fact that a majority of *Ap *genes have no known function poses the greatest challenge to interpreting these data. However, some things are clear: 1. Genes differentially transcribed between the human and tick cells disproportionately represent surface proteins (~45% compared to ~25% of all proteins) (Table 2). 2. There are genes, paralogs, and operons exclusively transcribed in the tick and the human cells, some of which may encode excellent vaccine candidates. 3. The particular paralogs of the *p44 *family of membrane proteins (114) expressed in a population of *Ap *may be identified by the elevated signal produced within the HVR of each as compared to silent paralogs. 4. Whole RNA isolated from *Ap *infected host cells can be used to reveal details of bacterial gene transcription, including that from anti-sense sequences. 5. Global transcription profiles can likely be generated for *Ap *in any host cells, and for all aspects of the cell infection cycle – cell binding, entry, growth, and escape – although some enrichment for bacteria or bacterial mRNA may be necessary. Coupling *Ap *transcription data with that of infected host cells will facilitate the discovery of *Ap *and host cell gene functions.

Having transcription data for all of an organism's DNA sequence allows a line graph display for both DNA strands parallel to an annotated map of the genome. This way one can readily see transcriptional behavior that may be less accessible through other analysis tools. For example, anti-sense transcription, and the variation in transcription profiles of genes – sloped, flat, horned, and reflected – may lead to important insights into *Ap *gene regulation, as well as for other intracellular organisms that subvert host cell processes for their own benefit.

## Authors' contributions

CN conceived of and carried out the tiling array study, led the array design effort, participated in data analysis, and drafted the manuscript. MH participated in the design of the study and the array, transformed the raw array data into interpretable formats, including adapting it to the Artemis annotation software, analyzed the data, and assisted in drafting the manuscript. RF participated in designing the study and the array, and assisted in the analysis and interpretation of the data, and in the preparation of the manuscript. BS performed the quantitative RT-PCR experiments. SG assisted with data analysis. AOC performed the *virB2 *sequence comparisons. TK and UM assisted in designing the study, the array, and in the preparation of the manuscript. All authors read and approved the final manuscript.

## Supplementary Material

Additional file 1 Materials and instructions for graphing transcript level data in Artemis Click here for file

Additional file 2 qRT-PCR primers Click here for file

Additional file 3 *Ap-*HL-60 vs. *Ap*-HMEC-1 differential transcription. 71 ORFs differentially transcribed (p ≤ 0.05) between *Ap*-HL-60 and *Ap*-HMEC-1 Click here for file

Additional file 4 Genes and operons with no detected transcripts. Artemis transcription plots showing examples of genes and an operon with no detectable transcript signal in any of the cell lines (Red: *Ap*-HL-60, Green: *Ap*-HMEC-1, Blue: *Ap*-ISE6; "smoothed" using a sliding window average of 5). (A) Genes *radC *(DNA repair) and *lipB *(lipoyl (octanoyl)-acyl carrier protein B). (B) An operon including loci *APH_0778 – APH_0783*. Click here for file

Additional file 5 Repeat-sequence-based sense and anti sense signal. Artemis plots showing sense and anti-sense transcript signal (shaded) within repeat sequences in two ORFs (Red: *Ap*-HL-60, Green: *Ap*-HMEC-1, Blue: *Ap*-ISE6; plots were "smoothed" by setting the sliding window average to 5). (A) *APH_0377 *(hypothetical). (B) *APH_0455 *(hypothetical). Click here for file

Additional file 6 Amino acid sequence alignments for the eight *virB2 *paralogs of *Ap*. (A) Pair-wise alignment of the eight *virB2 *amino acid sequences showing the percentages of identity and similarity between each. Identities rank from a high of 93% between tick cell expressed paralogs *APH_1133 *and *APH_1134*, and a low of 22% between non-expressed *APH_1136 *and human cell expressed *APH_1145*. (B) Multiple alignment showing higher identity and similarity between the C termini of paralogs, which contain the functional portion of the proteins. * Identical amino acids. · Conservative substitution. Click here for file

Additional file 7 Tick- and human-specific *Ap *operons. Artemis transcription plots showing *Ap *operons specific to the tick cell line (A, B) and the human cell lines (C). (Red: *Ap*-HL-60, Green: *Ap*-HMEC-1, Blue: *Ap*-ISE6.) (A) A tick operon (*APH_1380 *to *APH_1386*) that appears to include an unannotated ORF between *APH_1380 *and *APH_1383 *(in the same reading frame as *APH_1380*). Annotated loci *APH_1381 *and *APH_1382 *appear to be false ORFs. (B) Another tick-specific operon that includes loci *APH_0720 *to *APH_0726*. Loci *APH_0721 *and *APH_0722 *showed no transcription. (C) *Ap *genes transcribed only in the human cells: *APH_0837*, *APH_0838*, *APH_0839*, and *APH_0842*. Plots on panel A were smoothed using a sliding average of 5, and on panels B and C using a sliding average of 10. Click here for file

Additional file 8 Examples of other *Anaplasmataceae *bacteria with multiple *virB2 *loci. Examples of *Anaplasmataceae *bacteria with multiple *virB2 *loci. Click here for file

Additional file 9 *msp4 *transcription. Artemis transcription plots showing the position of the *msp4 *gene just downstream of anti-sense *p44 *sequences and its strong transcription by *Ap*-ISE6 (Red: *Ap*-HL-60, Green: *Ap*-HMEC-1, Blue: *Ap*-ISE6; plots were "smoothed" by setting the sliding window average to 5). "Read through" transcription of *msp4 *may be a source of anti-sense *p44 *transcripts in *Ap*-ISE6. Click here for file

## References

[B1] Dunning-Hotopp JC, Lin M, Madupu R, Crabtree J, Angiuoli SV, Eisen J, Seshadri R, Ren Q, Wu M, Utterback TR, Smith S, Lewis M, Khouri H, Zhang C, Niu H, Lin Q, Ohashi N, Zhi N, Nelson W, Brinkac LM, Dodson RJ, Rosovitz MJ, Sundaram J, Daugherty SC, Davidsen T, Durkin AS, Gwinn M, Haft DH, Selengut JD, Sullivan SA, Zafar N, Zhou L, Benahmed F, Forberger H, Halpin R, Mulligan S, Robinson J, White O, Rikihisa Y, Tettelin H (2006). Comparative genomics of emerging human ehrlichiosis agents. PLoS Genet.

[B2] Greig B, Asanovich KM, Armstrong PJ, Dumler JS (1996). Geographic, clinical, serologic, and molecular evidence of granulocytic ehrlichiosis, a likely zoonotic disease, in Minnesota and Wisconsin dogs. J Clin Microbiol.

[B3] Walls JJ, Greig B, Neitzel DF, Dumler JS (1997). Natural infection of small mammal species in Minnesota with the agent of human granulocytic ehrlichiosis. J Clin Microbiol.

[B4] Bullock PM, Ames TR, Robinson RA, Greig B, Mellencamp MA, Dumler JS (2000). Ehrlichia equi infection of horses from Minnesota and Wisconsin: detection of seroconversion and acute disease investigation. J Vet Intern Med.

[B5] Bayard-Mc Neeley M, Bansal A, Chowdhury I, Girao G, Small CB, Seiter K, Nelson J, Liveris D, Schwartz I, Mc Neeley DF, Wormser GP, Aguero-Rosenfeld ME (2004). *In vivo* and *in vitro* studies on Anaplasma phagocytophilum infection of the myeloid cells of a patient with chronic myelogenous leukaemia and human granulocytic ehrlichiosis. J Clin Pathol.

[B6] Klein MB, Nelson CM, Goodman JL (1997). Antibiotic susceptibility of the newly cultivated agent of human granulocytic ehrlichiosis: promising activity of quinolones and rifamycins. Antimicrob Agents Chemother.

[B7] Madigan JE, Gribble D (1987). Equine ehrlichiosis in northern California: 49 cases (1968–1981). J Am Vet Med Assoc.

[B8] Herron MJ, Ericson ME, Kurtti TJ, Munderloh UG (2005). The Interactions of Anaplasma phagocytophilum, Endothelial Cells, and Human Neutrophils. Ann N Y Acad Sci.

[B9] Klein MB, Miller JS, Nelson CM, Goodman JL (1997). Primary bone marrow progenitors of both granulocytic and monocytic lineages are susceptible to infection with the agent of human granulocytic ehrlichiosis. J Infect Dis.

[B10] Felek S, Telford S, Falco RC, Rikihisa Y (2004). Sequence analysis of p44 homologs expressed by Anaplasma phagocytophilum in infected ticks feeding on naive hosts and in mice infected by tick attachment. Infect Immun.

[B11] Holman MS, Caporale DA, Goldberg J, Lacombe E, Lubelczyk C, Rand PW, Smith RP (2004). Anaplasma phagocytophilum, Babesia microti, and Borrelia burgdorferi in Ixodes scapularis, southern coastal Maine. Emerg Infect Dis.

[B12] Sukumaran B, Narasimhan S, Anderson JF, Deponte K, Marcantonio N, Krishnan MN, Fish D, Telford SR, Kantor FS, Fikrig E (2006). An Ixodes scapularis protein required for survival of Anaplasma phagocytophilum in tick salivary glands. J Exp Med.

[B13] Munderloh UG, Jauron SD, Fingerle V, Leitritz L, Hayes SF, Hautman JM, Nelson CM, Huberty BW, Kurtti TJ, Ahlstrand GG, Greig B, Mellencamp MA, Goodman JL (1999). Invasion and intracellular development of the human granulocytic ehrlichiosis agent in tick cell culture. J Clin Microbiol.

[B14] Ades EW, Candal FJ, Swerlick RA, George VG, Summers S, Bosse DC, Lawley TJ (1992). HMEC-1: establishment of an immortalized human microvascular endothelial cell line. J Invest Dermatol.

[B15] Felsheim RF, Herron MJ, Nelson CM, Burkhardt NY, Barbet AF, Kurtti TJ, Munderloh UG (2006). Transformation of Anaplasma phagocytophilum. BMC Biotechnol.

[B16] Jauron SD, Nelson CM, Fingerle V, Ravyn MD, Goodman JL, Johnson RC, Lobentanzer R, Wilske B, Munderloh UG (2001). Host cell-specific expression of a p44 epitope by the human granulocytic ehrlichiosis agent. J Infect Dis.

[B17] Niu H, Rikihisa Y, Yamaguchi M, Ohashi N (2006). Differential expression of VirB9 and VirB6 during the life cycle of Anaplasma phagocytophilum in human leucocytes is associated with differential binding and avoidance of lysosome pathway. Cell Microbiol.

[B18] Ohashi N, Zhi N, Lin Q, Rikihisa Y (2002). Characterization and transcriptional analysis of gene clusters for a type IV secretion machinery in human granulocytic and monocytic ehrlichiosis agents. Infect Immun.

[B19] Lee HC, Goodman JL (2006). Anaplasma phagocytophilum causes global induction of antiapoptosis in human neutrophils. Genomics.

[B20] Carlyon JA, Chan WT, Galan J, Roos D, Fikrig E (2002). Repression of rac2 mRNA expression by Anaplasma phagocytophila is essential to the inhibition of superoxide production and bacterial proliferation. J Immunol.

[B21] de la Fuente J, Ayoubi P, Blouin EF, Almazan C, Naranjo V, Kocan KM (2005). Gene expression profiling of human promyelocytic cells in response to infection with Anaplasma phagocytophilum. Cell Microbiol.

[B22] Borjesson DL, Kobayashi SD, Whitney AR, Voyich JM, Argue CM, Deleo FR (2005). Insights into pathogen immune evasion mechanisms: Anaplasma phagocytophilum fails to induce an apoptosis differentiation program in human neutrophils. J Immunol.

[B23] Pedra JH, Sukumaran B, Carlyon JA, Berliner N, Fikrig E (2005). Modulation of NB4 promyelocytic leukemic cell machinery by Anaplasma phagocytophilum. Genomics.

[B24] Sukumaran B, Carlyon JA, Cai JL, Berliner N, Fikrig E (2005). Early transcriptional response of human neutrophils to Anaplasma phagocytophilum infection. Infect Immun.

[B25] Cerrina Fa, Blattnerb F, Huanga W, Huea Y, Greenc R, Singh-Gassonb S, Sussmanb M (2002). Biological lithography: development of a maskless microarray synthesizer for DNA chips. Microelectronic Engineering.

[B26] Liu XS (2007). Getting started in tiling microarray analysis. PLoS Comput Biol.

[B27] Mockler TC, Chan S, Sundaresan A, Chen H, Jacobsen SE, Ecker JR (2005). Applications of DNA tiling arrays for whole-genome analysis. Genomics.

[B28] Goodman JL, Nelson C, Vitale B, Madigan JE, Dumler JS, Kurtti TJ, Munderloh UG (1996). Direct cultivation of the causative agent of human granulocytic ehrlichiosis. N Engl J Med.

[B29] Munderloh UG, Lynch MJ, Herron MJ, Palmer AT, Kurtti TJ, Nelson RD, Goodman JL (2004). Infection of endothelial cells with Anaplasma marginale and A. phagocytophilum. Vet Microbiol.

[B30] Royce TE, Rozowsky JS, Luscombe NM, Emanuelsson O, Yu H, Zhu X, Snyder M, Gerstein MB (2006). Extrapolating traditional DNA microarray statistics to tiling and protein microarray technologies. Methods Enzymol.

[B31] Bendtsen JD, Kiemer L, Fausboll A, Brunak S (2005). Non-classical protein secretion in bacteria. BMC Microbiol.

[B32] Yu CS, Chen YC, Lu CH, Hwang JK (2006). Prediction of protein subcellular localization. Proteins.

[B33] Sarkar M, Troese MJ, Kearns SA, Yang T, Reneer DV, Carlyon JA (2008). Anaplasma phagocytophilum MSP2 (P44)-18 predominates and is modified into multiple isoforms in human myeloid cells. Infect Immun.

[B34] Wang X, Cheng Z, Zhang C, Kikuchi T, Rikihisa Y (2007). Anaplasma phagocytophilum p44 mRNA expression is differentially regulated in mammalian and tick host cells: involvement of the DNA binding protein ApxR. J Bacteriol.

[B35] Park J, Kim KJ, Choi KS, Grab DJ, Dumler JS (2004). Anaplasma phagocytophilum AnkA binds to granulocyte DNA and nuclear proteins. Cell Microbiol.

[B36] Caturegli P, Asanovich KM, Walls JJ, Bakken JS, Madigan JE, Popov VL, Dumler JS (2000). ankA: an Ehrlichia phagocytophila group gene encoding a cytoplasmic protein antigen with ankyrin repeats. Infect Immun.

[B37] Ijdo JW, Carlson AC, Kennedy EL (2007). Anaplasma phagocytophilum AnkA is tyrosine-phosphorylated at EPIYA motifs and recruits SHP-1 during early infection. Cell Microbiol.

[B38] Lin M, den Dulk-Ras A, Hooykaas PJ, Rikihisa Y (2007). Anaplasma phagocytophilum AnkA secreted by type IV secretion system is tyrosine phosphorylated by Abl-1 to facilitate infection. Cell Microbiol.

[B39] Bendtsen JD, Nielsen H, von Heijne G, Brunak S (2004). Improved prediction of signal peptides: SignalP 3.0. J Mol Biol.

[B40] Sonnhammer EL, von Heijne G, Krogh A (1998). A hidden Markov model for predicting transmembrane helices in protein sequences. Proc Int Conf Intell Syst Mol Biol.

[B41] den Hartigh AB, Sun YH, Sondervan D, Heuvelmans N, Reinders MO, Ficht TA, Tsolis RM (2004). Differential requirements for VirB1 and VirB2 during Brucella abortus infection. Infect Immun.

[B42] Lluch-Senar M, Vallmitjana M, Querol E, Pinol J (2007). A new promoterless reporter vector reveals antisense transcription in Mycoplasma genitalium. Microbiology.

[B43] Brantl S (2007). Regulatory mechanisms employed by cis-encoded antisense RNAs. Curr Opin Microbiol.

[B44] Lapidot M, Pilpel Y (2006). Genome-wide natural antisense transcription: coupling its regulation to its different regulatory mechanisms. EMBO Rep.

[B45] Timmons JA, Good L (2006). Does everything now make (anti)sense?. Biochem Soc Trans.

[B46] Zhi N, Ohashi N, Rikihisa Y (1999). Multiple p44 genes encoding major outer membrane proteins are expressed in the human granulocytic ehrlichiosis agent. J Biol Chem.

[B47] Lin Q, Zhang C, Rikihisa Y (2006). Analysis of involvement of the RecF pathway in p44 recombination in Anaplasma phagocytophilum and in Escherichia coli by using a plasmid carrying the p44 expression and p44 donor loci. Infect Immun.

