# Supplementary material for: Whole genome transcription profiling of Anaplasma phagocytophilum in human and tick host cells by tiling array analysis
Source: BMC Genomics. 2008 Jul 31;9:364. doi: 10.1186/1471-2164-9-364 (PMC2527338; doi:10.1186/1471-2164-9-364)
Supplement: Additional file 2 — qRT-PCR primers [file 1471-2164-9-364-S2.doc]

Additional file 2

| qRT-PCR target genes | | primer sequences | | |  | amplicon size |
| --- | --- | --- | --- | --- | --- | --- |
|  |  | |  |  |  |  |
| major surface protein 4 *(msp4*) | | F 5’ TGTTTCAGATCCTGCCAGCTTCAC 3’ | | | | 118 bp |
| (*APH_1240*) |  | | R 5’ AACTTCCACTCTAGCTCCGCCAAT 3’ | | |  |
|  |  | |  |  |  |  |
| outer membrane efflux protein | | F 5’ TGAGTGCTTTATCAGCCTACG 3’ | | | | 163 bp |
| (*APH_1110*) |  | | R 5’ GCTTCGGATTTTGCTTGTG 3’ | | |  |
|  |  | |  |  |  |  |
| major outer membrane protein (*omp-1A*) | | F 5’ GGGACAGGTTTAGATACTCGTTG 3’ | | | | 82 bp |
| (*APH_1359*) |  | | R 5’ TCGTTACGCAGCACCAAGA 3’ | | |  |
|  |  | |  |  |  |  |
| 60kDa chaperonin | | F 5’ GGAAGACCATTGCTCATCATAG 3’ | | | | 124 bp |
| (*APH_0240)* |  | | R 5’ TTCTGTCACCGAAACCAGG 3’ | | |  |
|  |  | |  |  |  |  |
| succinyl-CoA synthetase, beta subunit | | F 5’ TAACGCATCAAACTTCGGCT 3’ | | | | 144 bp |
| (*APH_1052)* |  | | R 5’ CCACCTTCACTGGAAAAGATAAC 3’ | | |  |
