# Supplementary figures and images for: Whole genome transcription profiling of Anaplasma phagocytophilum in human and tick host cells by tiling array analysis
Source: BMC Genomics. 2008 Jul 31;9:364. doi: 10.1186/1471-2164-9-364 (PMC2527338; doi:10.1186/1471-2164-9-364)

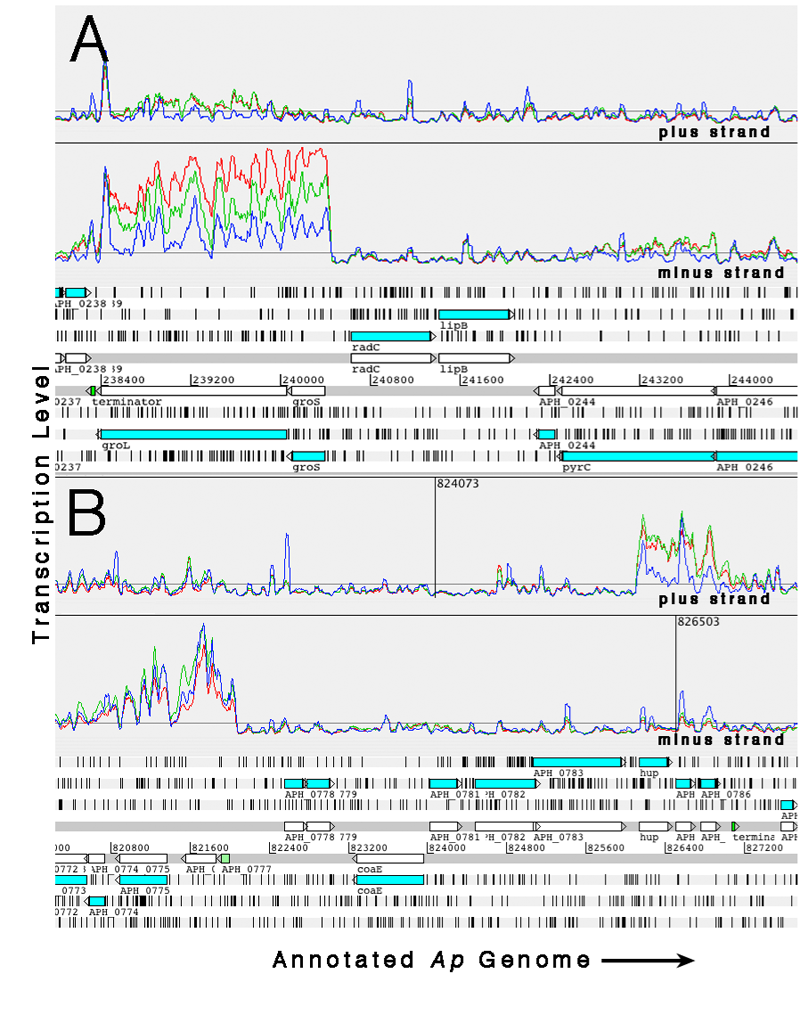

Supplement: Additional file 4 — Genes and operons with no detected transcripts. Artemis transcription plots showing examples of genes and an operon with no detectable transcript signal in any of the cell lines (Red: Ap-HL-60, Green: Ap-HMEC-1, Blue: Ap-ISE6; "smoothed" using a sliding window average of 5). (A) Genes radC (DNA repair) and lipB (lipoyl (octanoyl)-acyl carrier protein B). (B) An operon including loci APH_0778 – APH_0783. [file 1471-2164-9-364-S4.png]

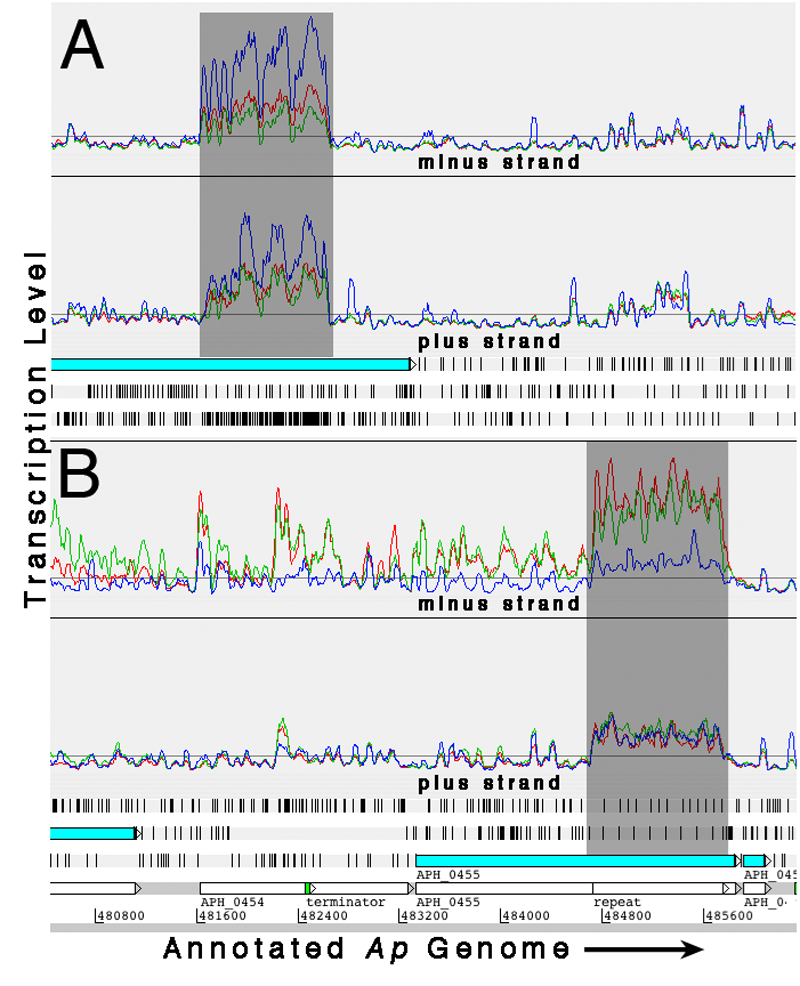

Supplement: Additional file 5 — Repeat-sequence-based sense and anti sense signal. Artemis plots showing sense and anti-sense transcript signal (shaded) within repeat sequences in two ORFs (Red: Ap-HL-60, Green: Ap-HMEC-1, Blue: Ap-ISE6; plots were "smoothed" by setting the sliding window average to 5). (A) APH_0377 (hypothetical). (B) APH_0455 (hypothetical). [file 1471-2164-9-364-S5.png]

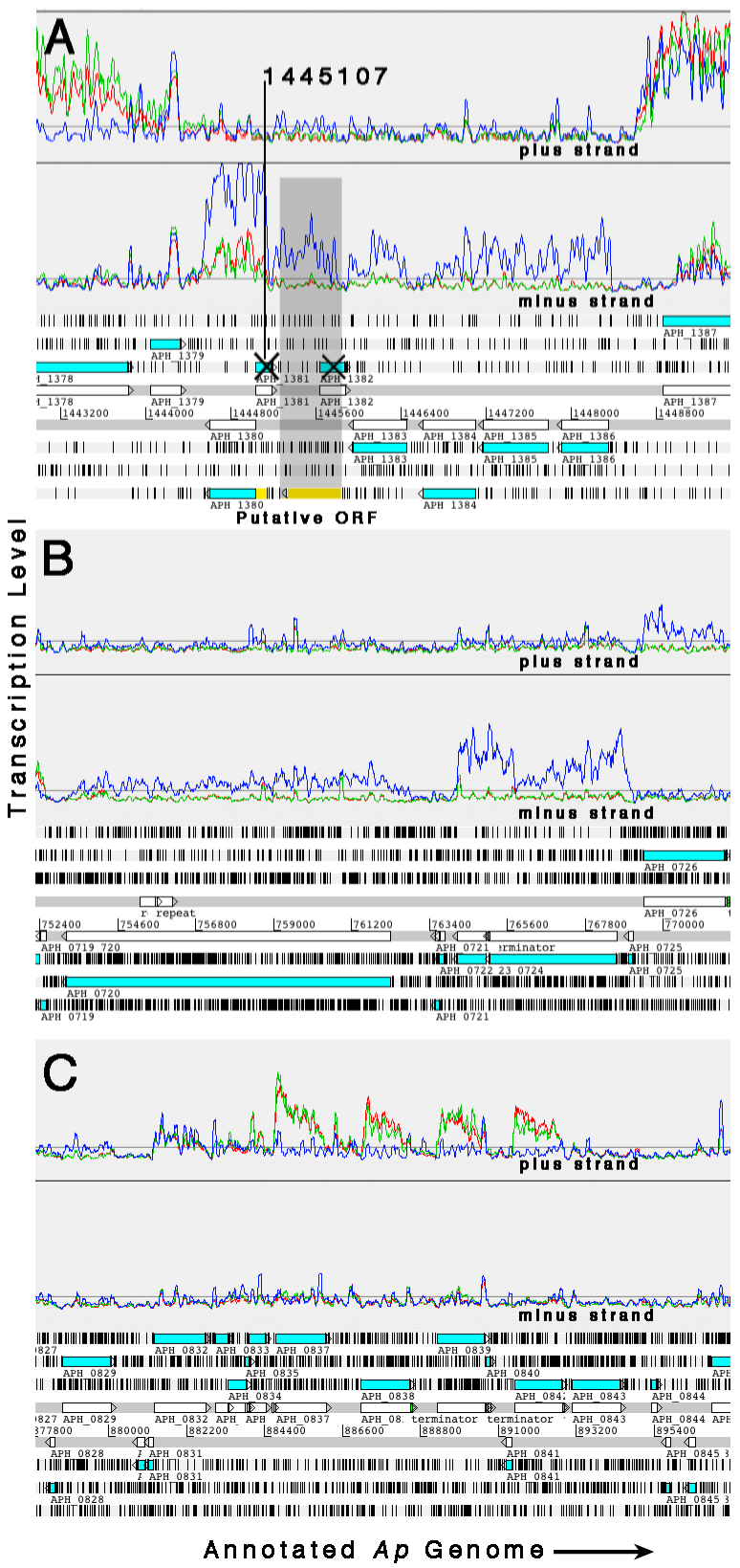

Supplement: Additional file 7 — Tick- and human-specific Ap operons. Artemis transcription plots showing Ap operons specific to the tick cell line (A, B) and the human cell lines (C). (Red: Ap-HL-60, Green: Ap-HMEC-1, Blue: Ap-ISE6.) (A) A tick operon (APH_1380 to APH_1386) that appears to include an unannotated ORF between APH_1380 and APH_1383 (in the same reading frame as APH_1380). Annotated loci APH_1381 and APH_1382 appear to be false ORFs. (B) Another tick-specific operon that includes loci APH_0720 to APH_0726. Loci APH_0721 and APH_0722 showed no transcription. (C) Ap genes transcribed only in the human cells: APH_0837, APH_0838, APH_0839, and APH_0842. Plots on panel A were smoothed using a sliding average of 5, and on panels B and C using a sliding average of 10. [file 1471-2164-9-364-S7.png]

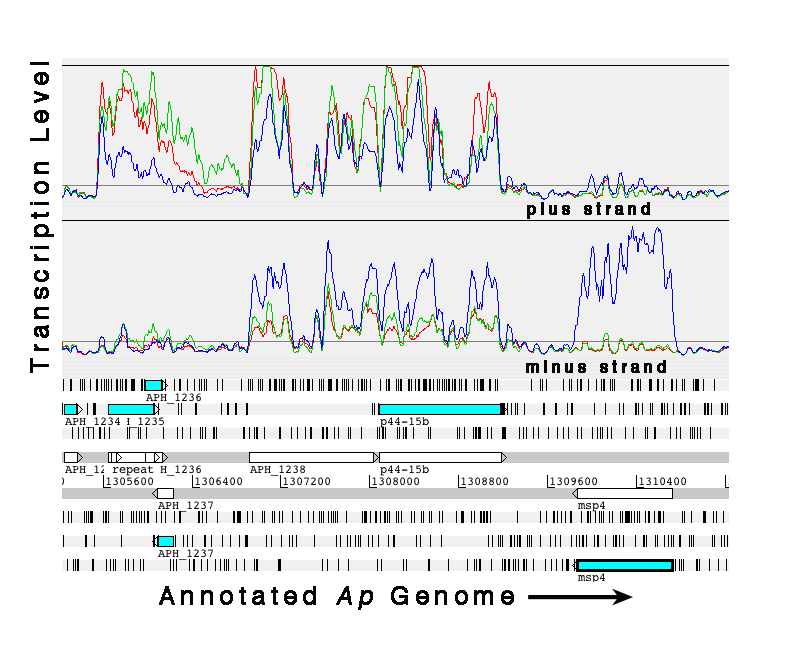

Supplement: Additional file 9 — msp4 transcription. Artemis transcription plots showing the position of the msp4 gene just downstream of anti-sense p44 sequences and its strong transcription by Ap-ISE6 (Red: Ap-HL-60, Green: Ap-HMEC-1, Blue: Ap-ISE6; plots were "smoothed" by setting the sliding window average to 5). "Read through" transcription of msp4 may be a source of anti-sense p44 transcripts in Ap-ISE6. [file 1471-2164-9-364-S9.png]
