# Supplementary material for: Whole genome transcription profiling of Anaplasma phagocytophilum in human and tick host cells by tiling array analysis
Source: BMC Genomics. 2008 Jul 31;9:364. doi: 10.1186/1471-2164-9-364 (PMC2527338; doi:10.1186/1471-2164-9-364)
Supplement: Additional file 6 — Amino acid sequence alignments for the eight virB2 paralogs of Ap. (A) Pair-wise alignment of the eight virB2 amino acid sequences showing the percentages of identity and similarity between each. Identities rank from a high of 93% between tick cell expressed paralogs APH_1133 and APH_1134, and a low of 22% between non-expressed APH_1136 and human cell expressed APH_1145. (B) Multiple alignment showing higher identity and similarity between the C termini of paralogs, which contain the functional portion of the proteins. * Identical amino acids. · Conservative substitution. [file 1471-2164-9-364-S6.doc]

**A**

ClustalW (v1.4) multiple sequence alignment

8 Sequences Aligned Alignment Score = 12031

Gaps Inserted = 16 Conserved Identities = 21

Pairwise Alignment Mode: Slow

Pairwise Alignment Parameters:

Open Gap Penalty = 35.0 Extend Gap Penalty = 0.8

Similarity Matrix: blosum

Multiple Alignment Parameters:

Open Gap Penalty = 15.0 Extend Gap Penalty = 0.3

Delay Divergent = 40% Gap Distance = 8

Similarity Matrix: blosum

Processing time: 0.5 seconds

1. APH_1130 vs. APH_1131

Aligned Length = 123 Gaps = 1

Identities = 63 (51%) Similarities = 25 (20%)

APH_1130 1 MFTNILRSCVISIIFFIFLILPAVSVSAAPVTHAAGDGEVISKVICNVVV 50

APH_1131 1 MAKVVRFFTSTVGMFLLLLLCSHGIASAAAAGTDHNG-VTAKVICNVVL 48

. ..* .. *. *.* . ...* . . * * .*******.

APH_1130 51 FVQRLGLPIMTGVILGSSIMAVFGRLAWPAIVMLVVFTAIFFGAGKLISK 100

APH_1131 49 FVQKLGLPIMTGVILGSSVMAIFGRLAWPSIAMLIVFTAIFFGSSKIIGK 98

***.**************.**.*******.* **.********. *.* *

APH_1130 101 FAGGISELGAEDFDCRVLAGKNI 123

APH_1131 99 FAQGVGEWEAEKFDCKDIKAG 119

** *. * ** ***. .

2. APH_1130 vs. APH_1134

Aligned Length = 123 Gaps = 0

Identities = 68 (55%) Similarities = 21 (17%)

APH_1130 1 MFTNILRSCVISIIFFIFLILPAVSVSAAPVTHAAGDGEVISKVICNVVV 50

APH_1134 1 MAKIVRFFTSTAGMFLLLLLCSQGVAAGASANDEHKKEETSKVICNVVL 49

. *.* . *. *.* . *.* .. * ********.

APH_1130 51 FVQRLGLPIMTGVILGSSIMAVFGRLAWPAIVMLVVFTAIFFGAGKLISK 100

APH_1134 50 FAQKLGLPIMTGVILGSSVMAIFGRLAWPAIAMLIVFTAIFFGSSKIISK 99

* *.**************.**.********* **.********. *.***

APH_1130 101 FAGGISELGAEDFDCRVLAGKNI 123

APH_1134 100 FANGVGEIKANDFDCKEVAEK 120

**.*. *. * ****. .* *

3. APH_1130 vs. APH_1144

Aligned Length = 129 Gaps = 1

Identities = 64 (52%) Similarities = 21 (17%)

APH_1130 1 MFTNILRSCVISIIFFIFLILPAVSVSAAPVTHAAGDGEVISKVICNVVV 50

APH_1144 1 MFGLTRFMAVLALVVALVGFGTSAFASTTGSDDVAAKVICNVVV 44

. . . *.. * .. . ..* .* .********

APH_1130 51 FVQRLGLPIMTGVILGSSIMAVFGRLAWPAIVMLVVFTAIFFGAGKLISK 100

APH_1144 45 FVQRLGLPIMTGVILGASIMAIFGKLAWAAIVMLVVFTAIFFGAGKLIQK 94

****************.****.**.*** ******************* *

APH_1130 101 FAGGIS----ELGAEDFDCRVLAGKNI 123

APH_1144 95 FAAGVGSDIIGGNAESFECKGNGATTLSS 123

** *. .** *.*. ..

4. APH_1130 vs. APH_1132

Aligned Length = 126 Gaps = 1

Identities = 66 (53%) Similarities = 22 (17%)

APH_1130 1 MFTNILRSCVISIIFFIFLILPAVSVSAAPVTHAAGDGEVISKVICNVVV 50

APH_1132 1 MAKIVRFFTSTVGMFLLLLLCSHGIASAAAAGTDHNG-VTAKVICNVVL 48

. *.* .. *. *.* . ...* . . * * .*******.

APH_1130 51 FVQRLGLPIMTGVILGSSIMAVFGRLAWPAIVMLVVFTAIFFGAGKLISK 100

APH_1132 49 FVQKLGLPIMTGVILGSSVMAIFGRLAWPAIAMLIVFTAIFFGSSKIIGK 98

***.**************.**.********* **.********. *.* *

APH_1130 101 FAGGISELGAEDFDCRVLAGKNI 123

APH_1132 99 FAKGIGELDADNFDCSKVQAEESSSV 124

** ** ** *. *** .

5. APH_1130 vs. APH_1133

Aligned Length = 123 Gaps = 0

Identities = 64 (52%) Similarities = 23 (18%)

APH_1130 1 MFTNILRSCVISIIFFIFLILPAVSVSAAPVTHAAGDGEVISKVICNVVV 50

APH_1133 1 MAKIVRFFTSTAGMFLLLLLCSQGVAAGASANDEHKKEETSKVICNVVL 49

. *.* . *. *.* . *.* .. * ********.

APH_1130 51 FVQRLGLPIMTGVILGSSIMAVFGRLAWPAIVMLVVFTAIFFGAGKLISK 100

APH_1133 50 FAQKLGLPIMTGVILGSSVMAIFGRLAWPAIAMLIVFTAIFFGSSKIIGK 99

* *.**************.**.********* **.********. *.* *

APH_1130 101 FAGGISELGAEDFDCRVLAGKNI 123

APH_1133 100 FANGVGDLKATEFDCKEVTK 119

**.*. .* * .***. ..

6. APH_1130 vs. APH_1145

Aligned Length = 130 Gaps = 2

Identities = 40 (31%) Similarities = 26 (20%)

APH_1130 1 MFTNILRSCVISIIFFIFLILPAVSVS----AAPVTHAAGDGEVISK 43

APH_1145 1 MMSNLTGFVAVLSVIMMFGVAGAIDACGVEPTAEKDHTVAVPIKGDVAVK 50

*. . . ..* . . * * *.* *

APH_1130 44 VICNVVVFVQRLGLPIMTGVILGSSIMAVFGRLAWPAIVMLVVFTAIFFG 93

APH_1145 51 SVSGVLQTVRRFCLPVMIGVVSGAVIITVFGRSAWFAIAMLIVFSCIFLG 100

...*. *.* **.* **. *. *..**** ** ** **.**..** *

APH_1130 94 AGKLISKFAGGISELGAEDFDCRVLAGKNI 123

APH_1145 101 GSEFIQKFTEGVG-DSAGTKHSRVIASRL 128

* **. *. * .**.* .

7. APH_1130 vs. APH_1136

Aligned Length = 151 Gaps = 1

Identities = 58 (40%) Similarities = 21 (14%)

APH_1130 1 MFTNILRSCVISIIFFIFLILPAVSVS---- 27

APH_1136 1 MDTQGRAIAEDRRSFARTFFNKKVFFLIIQGSLFFVLLLILDEAYAGVAE 50

. . .**. *.. . .

APH_1130 28 -----AAPVTHAAGDGEVISKVICNVVVFVQRLGLPIMTGVILGSSIMAV 72

APH_1136 51 SNLFPAVAQHGSATNEDVTSKVICNVVKFVRGIGLPIMTGVILGSSVMAI 100

* .* .* ******** **. .*************.**.

APH_1130 73 FGRLAWPAIVMLVVFTAIFFGAGKLISKFAGGISELGAEDFDCRVLAGKN 122

APH_1136 101 FGRLAWPAIAALVIFTAVFFGAEKVISKFTDGISVMQTGNCDTI 144

********* **.***.**** *.****. *** . . *.

APH_1130 123 I 123

APH_1136 145 144

8. APH_1131 vs. APH_1134

Aligned Length = 120 Gaps = 1

Identities = 92 (76%) Similarities = 12 (10%)

APH_1131 1 MAKVVRFFTSTVGMFLLLLLCSHGIASAAAAGTDHNG-VTAKVICNVVLF 49

APH_1134 1 MAKIVRFFTSTAGMFLLLLLCSQGVAAGASANDEHKKEETSKVICNVVLF 50

***.******* **********.*.*. *.*. .* *.*********

APH_1131 50 VQKLGLPIMTGVILGSSVMAIFGRLAWPSIAMLIVFTAIFFGSSKIIGKF 99

APH_1134 51 AQKLGLPIMTGVILGSSVMAIFGRLAWPAIAMLIVFTAIFFGSSKIISKF 100

***************************.****************** **

APH_1131 100 AQGVGEWEAEKFDCKDIKAG 119

APH_1134 101 ANGVGEIKANDFDCKEVAEK 120

*.**** * ****..

9. APH_1131 vs. APH_1144

Aligned Length = 128 Gaps = 2

Identities = 65 (52%) Similarities = 17 (13%)

APH_1131 1 MAKVVRFFTSTVGMFLLLLLCSHGIASAAAAGTDHNG-VTAKVICNVVLF 49

APH_1144 1 MFGLTRFMAVLALVVALVGFGTSAFASTTGSDDVAAKVICNVVVF 45

* . * *... * ..* * * *.********.*

APH_1131 50 VQKLGLPIMTGVILGSSVMAIFGRLAWPSIAMLIVFTAIFFGSSKIIGKF 99

APH_1144 46 VQRLGLPIMTGVILGASIMAIFGKLAWAAIVMLVVFTAIFFGAGKLIQKF 95

**.************.*.*****.*** .* **.********. *.* **

APH_1131 100 AQGVG----EWEAEKFDCKDIKAG 119

APH_1144 96 AAGVGSDIIGGNAESFECKGNGATTLSS 123

* *** ** *.** *

10. APH_1131 vs. APH_1132

Aligned Length = 124 Gaps = 0

Identities = 106 (85%) Similarities = 8 (6%)

APH_1131 1 MAKVVRFFTSTVGMFLLLLLCSHGIASAAAAGTDHNGVTAKVICNVVLFV 50

APH_1132 1 MAKIVRFFTSTVGMFLLLLLCSHGIASAAAAGTDHNGVTAKVICNVVLFV 50

***.**********************************************

APH_1131 51 QKLGLPIMTGVILGSSVMAIFGRLAWPSIAMLIVFTAIFFGSSKIIGKFA 100

APH_1132 51 QKLGLPIMTGVILGSSVMAIFGRLAWPAIAMLIVFTAIFFGSSKIIGKFA 100

***************************.**********************

APH_1131 101 QGVGEWEAEKFDCKDIKAG 119

APH_1132 101 KGIGELDADNFDCSKVQAEESSSV 124

.*.** .*. *** ..*

11. APH_1131 vs. APH_1133

Aligned Length = 120 Gaps = 1

Identities = 92 (77%) Similarities = 13 (10%)

APH_1131 1 MAKVVRFFTSTVGMFLLLLLCSHGIASAAAAGTDHNG-VTAKVICNVVLF 49

APH_1133 1 MAKIVRFFTSTAGMFLLLLLCSQGVAAGASANDEHKKEETSKVICNVVLF 50

***.******* **********.*.*. *.*. .* *.*********

APH_1131 50 VQKLGLPIMTGVILGSSVMAIFGRLAWPSIAMLIVFTAIFFGSSKIIGKF 99

APH_1133 51 AQKLGLPIMTGVILGSSVMAIFGRLAWPAIAMLIVFTAIFFGSSKIIGKF 100

***************************.*********************

APH_1131 100 AQGVGEWEAEKFDCKDIKAG 119

APH_1133 101 ANGVGDLKATEFDCKEVTK 119

*.***. * ****..

12. APH_1131 vs. APH_1145

Aligned Length = 129 Gaps = 3

Identities = 37 (28%) Similarities = 31 (24%)

APH_1131 1 MAKVVRFFTSTVGMFLLLLLCSHGIA----SAAAAGTDHNG-VTAK 41

APH_1145 1 MMSNLTGFVAVLSVIMMFGVAGAIDACGVEPTAEKDHTVAVPIKGDVAVK 50

.. * . . . . . *. . * *. *

APH_1131 42 VICNVVLFVQKLGLPIMTGVILGSSVMAIFGRLAWPSIAMLIVFTAIFFG 91

APH_1145 51 SVSGVLQTVRRFCLPVMIGVVSGAVIITVFGRSAWFAIAMLIVFSCIFLG 100

...*. *.. **.* **. *. ....*** ** .*******..** *

APH_1131 92 SSKIIGKFAQGVGEWEAEKFDCKDIKAG 119

APH_1145 101 GSEFIQKFTEGVG-DSAGTKHSRVIASRL 128

* * **..*** * .. * .

13. APH_1131 vs. APH_1136

Aligned Length = 149 Gaps = 2

Identities = 60 (41%) Similarities = 18 (12%)

APH_1131 1 MAKVVRFFTSTVGMFLLLLLCSHGIA---- 26

APH_1136 1 MDTQGRAIAEDRRSFARTFFNKKVFFLIIQGSLFFVLLLILDEAYAGVAE 50

** *.***. *

APH_1131 27 -----SAAAAGTDHNG-VTAKVICNVVLFVQKLGLPIMTGVILGSSVMAI 70

APH_1136 51 SNLFPAVAQHGSATNEDVTSKVICNVVKFVRGIGLPIMTGVILGSSVMAI 100

. * *. * **.******* **. .*****************

APH_1131 71 FGRLAWPSIAMLIVFTAIFFGSSKIIGKFAQGVGEWEAEKFDCKDIKAG 119

APH_1136 101 FGRLAWPAIAALVIFTAVFFGAEKVISKFTDGISVMQTGNCDTI 144

*******.** *..***.***. *.* **. *. .. *.

14. APH_1134 vs. APH_1144

Aligned Length = 128 Gaps = 1

Identities = 60 (48%) Similarities = 17 (13%)

APH_1134 1 MAKIVRFFTSTAGMFLLLLLCSQGVAAGASANDEHKKEETSKVICNVVLF 50

APH_1144 1 MFGLTRFMAVLALVVALVGFGTSAFASTTGSDDVAAKVICNVVVF 45

* . * *... * . * * . ..*******.*

APH_1134 51 AQKLGLPIMTGVILGSSVMAIFGRLAWPAIAMLIVFTAIFFGSSKIISKF 100

APH_1144 46 VQRLGLPIMTGVILGASIMAIFGKLAWAAIVMLVVFTAIFFGAGKLIQKF 95

*.************.*.*****.*** ** **.********. *.* **

APH_1134 101 ANGVG----EIKANDFDCKEVAEK 120

APH_1144 96 AAGVGSDIIGGNAESFECKGNGATTLSS 123

* *** * *.**

15. APH_1134 vs. APH_1132

Aligned Length = 125 Gaps = 1

Identities = 93 (75%) Similarities = 9 (7%)

APH_1134 1 MAKIVRFFTSTAGMFLLLLLCSQGVAAGASANDEHKKEETSKVICNVVLF 50

APH_1132 1 MAKIVRFFTSTVGMFLLLLLCSHGIASAAAAGTDHNG-VTAKVICNVVLF 49

*********** **********.*.*. *.*. .* *.*********

APH_1134 51 AQKLGLPIMTGVILGSSVMAIFGRLAWPAIAMLIVFTAIFFGSSKIISKF 100

APH_1132 50 VQKLGLPIMTGVILGSSVMAIFGRLAWPAIAMLIVFTAIFFGSSKIIGKF 99

********************************************** **

APH_1134 101 ANGVGEIKANDFDCKEVAEK 120

APH_1132 100 AKGIGELDADNFDCSKVQAEESSSV 124

* *.**. * *** *

16. APH_1134 vs. APH_1133

Aligned Length = 120 Gaps = 0

Identities = 112 (93%) Similarities = 5 (4%)

APH_1134 1 MAKIVRFFTSTAGMFLLLLLCSQGVAAGASANDEHKKEETSKVICNVVLF 50

APH_1133 1 MAKIVRFFTSTAGMFLLLLLCSQGVAAGASANDEHKKEETSKVICNVVLF 50

**************************************************

APH_1134 51 AQKLGLPIMTGVILGSSVMAIFGRLAWPAIAMLIVFTAIFFGSSKIISKF 100

APH_1133 51 AQKLGLPIMTGVILGSSVMAIFGRLAWPAIAMLIVFTAIFFGSSKIIGKF 100

*********************************************** **

APH_1134 101 ANGVGEIKANDFDCKEVAEK 120

APH_1133 101 ANGVGDLKATEFDCKEVTK 119

*****..**..******.

17. APH_1134 vs. APH_1145

Aligned Length = 129 Gaps = 2

Identities = 37 (28%) Similarities = 30 (23%)

APH_1134 1 MAKIVRFFTSTAGMFLLLLLCSQGVA----AGASANDEHKKEETSK 42

APH_1145 1 MMSNLTGFVAVLSVIMMFGVAGAIDACGVEPTAEKDHTVAVPIKGDVAVK 50

.. * . . . . ** . * . . *

APH_1134 43 VICNVVLFAQKLGLPIMTGVILGSSVMAIFGRLAWPAIAMLIVFTAIFFG 92

APH_1145 51 SVSGVLQTVRRFCLPVMIGVVSGAVIITVFGRSAWFAIAMLIVFSCIFLG 100

...*. .. **.* **. *. ....*** ** ********..** *

APH_1134 93 SSKIISKFANGVGEIKANDFDCKEVAEK 120

APH_1145 101 GSEFIQKFTEGVG-DSAGTKHSRVIASRL 128

* * **. *** *. .. .* .

18. APH_1134 vs. APH_1136

Aligned Length = 149 Gaps = 1

Identities = 59 (40%) Similarities = 21 (14%)

APH_1134 1 MAKIVRFFTSTAGMFLLLLLCSQGVA---- 26

APH_1136 1 MDTQGRAIAEDRRSFARTFFNKKVFFLIIQGSLFFVLLLILDEAYAGVAE 50

*. . *.***. . *

APH_1134 27 -----AGASANDEHKKEETSKVICNVVLFAQKLGLPIMTGVILGSSVMAI 71

APH_1136 51 SNLFPAVAQHGSATNEDVTSKVICNVVKFVRGIGLPIMTGVILGSSVMAI 100

* * . . ********* * . .*****************

APH_1134 72 FGRLAWPAIAMLIVFTAIFFGSSKIISKFANGVGEIKANDFDCKEVAEK 120

APH_1136 101 FGRLAWPAIAALVIFTAVFFGAEKVISKFTDGISVMQTGNCDTI 144

********** *..***.***. *.****. *. .... *.

19. APH_1144 vs. APH_1132

Aligned Length = 129 Gaps = 2

Identities = 65 (52%) Similarities = 18 (14%)

APH_1144 1 MFGLTRFMAVLALVVALVGFGTSAFASTTGSDDVAAKVICNVVVF 45

APH_1132 1 MAKIVRFFTSTVGMFLLLLLCSHGIASAAAAGTDHNG-VTAKVICNVVLF 49

* . * *... * ..* * * *.********.*

APH_1144 46 VQRLGLPIMTGVILGASIMAIFGKLAWAAIVMLVVFTAIFFGAGKLIQKF 95

APH_1132 50 VQKLGLPIMTGVILGSSVMAIFGRLAWPAIAMLIVFTAIFFGSSKIIGKF 99

**.************.*.*****.*** ** **.********. *.* **

APH_1144 96 AAGVGSDIIGGNAESFECKGNGATTLSS 123

APH_1132 100 AKGIG----ELDADNFDCSKVQAEESSSV 124

* *.* *. *.* * **

20. APH_1144 vs. APH_1133

Aligned Length = 128 Gaps = 1

Identities = 60 (48%) Similarities = 17 (13%)

APH_1144 1 MFGLTRFMAVLALVVALVGFGTSAFASTTGSDDVAAKVICNVVVF 45

APH_1133 1 MAKIVRFFTSTAGMFLLLLLCSQGVAAGASANDEHKKEETSKVICNVVLF 50

* . * *... * . * * . ..*******.*

APH_1144 46 VQRLGLPIMTGVILGASIMAIFGKLAWAAIVMLVVFTAIFFGAGKLIQKF 95

APH_1133 51 AQKLGLPIMTGVILGSSVMAIFGRLAWPAIAMLIVFTAIFFGSSKIIGKF 100

*.************.*.*****.*** ** **.********. *.* **

APH_1144 96 AAGVGSDIIGGNAESFECKGNGATTLSS 123

APH_1133 101 ANGVG----DLKATEFDCKEVTK 119

* *** * *.**

21. APH_1144 vs. APH_1145

Aligned Length = 136 Gaps = 2

Identities = 39 (30%) Similarities = 22 (17%)

APH_1144 1 MFGLTRFMAVLALVVALVGFG----TSAFASTTGSDDVAAK 37

APH_1145 1 MMSNLTGFVAVLSVIMMFGVAGAIDACGVEPTAEKDHTVAVPIKGDVAVK 50

* .* . * . *** *

APH_1144 38 VICNVVVFVQRLGLPIMTGVILGASIMAIFGKLAWAAIVMLVVFTAIFFG 87

APH_1145 51 SVSGVLQTVRRFCLPVMIGVVSGAVIITVFGRSAWFAIAMLIVFSCIFLG 100

...*. *.* **.* **. ** *...**. ** ** **.**..** *

APH_1144 88 AGKLIQKFAAGVGSDIIGGNAESFECKGNGATTLSS 123

APH_1145 101 GSEFIQKFTEGVG-----DSAGTKHSRVIASRL 128

****. *** * . .. .

22. APH_1144 vs. APH_1136

Aligned Length = 157 Gaps = 2

Identities = 49 (34%) Similarities = 26 (18%)

APH_1144 1 MFGLTRFMAVLALVVALVGFG---- 21

APH_1136 1 MDTQGRAIAEDRRSFARTFFNKKVFFLIIQGSLFFVLLLILDEAYAGVAE 50

. . . *.. .

APH_1144 22 -----TSAFASTTGSDDVAAKVICNVVVFVQRLGLPIMTGVILGASIMAI 66

APH_1136 51 SNLFPAVAQHGSATNEDVTSKVICNVVKFVRGIGLPIMTGVILGSSVMAI 100

. * .. .**..******* **. .***********.*.***

APH_1144 67 FGKLAWAAIVMLVVFTAIFFGAGKLIQKFAAGVGSDIIGGNAESFECKGN 116

APH_1136 101 FGRLAWPAIAALVIFTAVFFGAEKVISKFTDGIS----VMQTGNCDTI 144

**.*** ** **.***.**** *.* **. *. .. ..

APH_1144 117 GATTLSS 123

APH_1136 145 144

23. APH_1132 vs. APH_1133

Aligned Length = 125 Gaps = 1

Identities = 94 (75%) Similarities = 9 (7%)

APH_1132 1 MAKIVRFFTSTVGMFLLLLLCSHGIASAAAAGTDHNG-VTAKVICNVVLF 49

APH_1133 1 MAKIVRFFTSTAGMFLLLLLCSQGVAAGASANDEHKKEETSKVICNVVLF 50

*********** **********.*.*. *.*. .* *.*********

APH_1132 50 VQKLGLPIMTGVILGSSVMAIFGRLAWPAIAMLIVFTAIFFGSSKIIGKF 99

APH_1133 51 AQKLGLPIMTGVILGSSVMAIFGRLAWPAIAMLIVFTAIFFGSSKIIGKF 100

*************************************************

APH_1132 100 AKGIGELDADNFDCSKVQAEESSSV 124

APH_1133 101 ANGVGDLKATEFDCKEVTK 119

* *.*.* * *** *

24. APH_1132 vs. APH_1145

Aligned Length = 133 Gaps = 3

Identities = 36 (28%) Similarities = 31 (24%)

APH_1132 1 MAKIVRFFTSTVGMFLLLLLCSHGIA----SAAAAGTDHNG-VTAK 41

APH_1145 1 MMSNLTGFVAVLSVIMMFGVAGAIDACGVEPTAEKDHTVAVPIKGDVAVK 50

.. * . . . . . *. . * *. *

APH_1132 42 VICNVVLFVQKLGLPIMTGVILGSSVMAIFGRLAWPAIAMLIVFTAIFFG 91

APH_1145 51 SVSGVLQTVRRFCLPVMIGVVSGAVIITVFGRSAWFAIAMLIVFSCIFLG 100

...*. *.. **.* **. *. ....*** ** ********..** *

APH_1132 92 SSKIIGKFAKGIGELDADNFDCSKVQAEESSSV 124

APH_1145 101 GSEFIQKFTEGVG-DSAGTKHSRVIASRL 128

* * **. *.* * . . . .

25. APH_1132 vs. APH_1136

Aligned Length = 154 Gaps = 2

Identities = 62 (43%) Similarities = 17 (11%)

APH_1132 1 MAKIVRFFTSTVGMFLLLLLCSHGIA---- 26

APH_1136 1 MDTQGRAIAEDRRSFARTFFNKKVFFLIIQGSLFFVLLLILDEAYAGVAE 50

*. *.***. *

APH_1132 27 -----SAAAAGTDHNG-VTAKVICNVVLFVQKLGLPIMTGVILGSSVMAI 70

APH_1136 51 SNLFPAVAQHGSATNEDVTSKVICNVVKFVRGIGLPIMTGVILGSSVMAI 100

. * *. * **.******* **. .*****************

APH_1132 71 FGRLAWPAIAMLIVFTAIFFGSSKIIGKFAKGIGELDADNFDCSKVQAEE 120

APH_1136 101 FGRLAWPAIAALVIFTAVFFGAEKVISKFTDGISVMQTGNCDTI 144

********** *..***.***. *.* **. ** . . * *.

APH_1132 121 SSSV 124

APH_1136 145 144

26. APH_1133 vs. APH_1145

Aligned Length = 129 Gaps = 2

Identities = 36 (28%) Similarities = 29 (22%)

APH_1133 1 MAKIVRFFTSTAGMFLLLLLCSQGVA----AGASANDEHKKEETSK 42

APH_1145 1 MMSNLTGFVAVLSVIMMFGVAGAIDACGVEPTAEKDHTVAVPIKGDVAVK 50

.. * . . . . ** . * . . *

APH_1133 43 VICNVVLFAQKLGLPIMTGVILGSSVMAIFGRLAWPAIAMLIVFTAIFFG 92

APH_1145 51 SVSGVLQTVRRFCLPVMIGVVSGAVIITVFGRSAWFAIAMLIVFSCIFLG 100

...*. .. **.* **. *. ....*** ** ********..** *

APH_1133 93 SSKIIGKFANGVGDLKATEFDCKEVTK 119

APH_1145 101 GSEFIQKFTEGVG-DSAGTKHSRVIASRL 128

* * **. *** * .. ..

27. APH_1133 vs. APH_1136

Aligned Length = 148 Gaps = 1

Identities = 58 (40%) Similarities = 20 (13%)

APH_1133 1 MAKIVRFFTSTAGMFLLLLLCSQGVA---- 26

APH_1136 1 MDTQGRAIAEDRRSFARTFFNKKVFFLIIQGSLFFVLLLILDEAYAGVAE 50

*. . *.***. . *

APH_1133 27 -----AGASANDEHKKEETSKVICNVVLFAQKLGLPIMTGVILGSSVMAI 71

APH_1136 51 SNLFPAVAQHGSATNEDVTSKVICNVVKFVRGIGLPIMTGVILGSSVMAI 100

* * . . ********* * . .*****************

APH_1133 72 FGRLAWPAIAMLIVFTAIFFGSSKIIGKFANGVGDLKATEFDCKEVTK 119

APH_1136 101 FGRLAWPAIAALVIFTAVFFGAEKVISKFTDGISVMQTGNCDTI 144

********** *..***.***. *.* **. *. ... *.

28. APH_1145 vs. APH_1136

Aligned Length = 150 Gaps = 2

Identities = 33 (22%) Similarities = 26 (18%)

APH_1145 1 MMSNLTGFVAVLSVIMMFGVAGAIDACGVEP--- 31

APH_1136 1 MDTQGRAIAEDRRSFARTFFNKKVFFLIIQGSLFFVLLLILDEAYAGVAE 50

.. .* * *

APH_1145 32 --TAEKDHTVAVPIKGDVAVKSVSGVLQTVRRFCLPVMIGVVSGAVIITV 79

APH_1136 51 SNLFPAVAQHGSATNEDVTSKVICNVVKFVRGIGLPIMTGVILGSSVMAI 100

**. * ...*.. ** **.* **. *. ....

APH_1145 80 FGRSAWFAIAMLIVFSCIFLGGSEFIQKFTEGVG-DSAGTKHSRVIASRL 128

APH_1136 101 FGRLAWPAIAALVIFTAVFFGAEKVISKFTDGISVMQTGNCDTI 144

*** ** *** *..*...* * * ***.*. .*. .

**B**

ClustalW (v1.4) multiple sequence alignment

8 Sequences Aligned Alignment Score = 12031

Gaps Inserted = 16 Conserved Identities = 21

Pairwise Alignment Mode: Slow

Pairwise Alignment Parameters:

Open Gap Penalty = 35.0 Extend Gap Penalty = 0.8

Similarity Matrix: blosum

Multiple Alignment Parameters:

Open Gap Penalty = 15.0 Extend Gap Penalty = 0.3

Delay Divergent = 40% Gap Distance = 8

Similarity Matrix: blosum

Processing time: 0.5 seconds

APH_1130 1 MFTNILRSCVISIIFFIFLILPAVSVS---- 27

APH_1131 1 MAKVVRFFTSTVGMFLLLLLCSHGIA---- 26

APH_1134 1 MAKIVRFFTSTAGMFLLLLLCSQGVA---- 26

APH_1144 1 MFGLTRFMAVLALVVALVGFG---- 21

APH_1132 1 MAKIVRFFTSTVGMFLLLLLCSHGIA---- 26

APH_1133 1 MAKIVRFFTSTAGMFLLLLLCSQGVA---- 26

APH_1145 1 MMSNLTGFVAVLSVIMMFGVAGAIDACGVEP--- 31

APH_1136 1 MDTQGRAIAEDRRSFARTFFNKKVFFLIIQGSLFFVLLLILDEAYAGVAE 50

. .

APH_1130 28 -----AAPVTHAAGDGEVISKVICNVVVFVQRLGLPIMTGVILGSSIMAV 72

APH_1131 27 -----SAAAAGTDHNG-VTAKVICNVVLFVQKLGLPIMTGVILGSSVMAI 70

APH_1134 27 -----AGASANDEHKKEETSKVICNVVLFAQKLGLPIMTGVILGSSVMAI 71

APH_1144 22 -----TSAFASTTGSDDVAAKVICNVVVFVQRLGLPIMTGVILGASIMAI 66

APH_1132 27 -----SAAAAGTDHNG-VTAKVICNVVLFVQKLGLPIMTGVILGSSVMAI 70

APH_1133 27 -----AGASANDEHKKEETSKVICNVVLFAQKLGLPIMTGVILGSSVMAI 71

APH_1145 32 --TAEKDHTVAVPIKGDVAVKSVSGVLQTVRRFCLPVMIGVVSGAVIITV 79

APH_1136 51 SNLFPAVAQHGSATNEDVTSKVICNVVKFVRGIGLPIMTGVILGSSVMAI 100

* ...*. . **.* **. *. ....

APH_1130 73 FGRLAWPAIVMLVVFTAIFFGAGKLISKFAGGIS----ELGAEDFDCRVL 118

APH_1131 71 FGRLAWPSIAMLIVFTAIFFGSSKIIGKFAQGVG----EWEAEKFDCKDI 116

APH_1134 72 FGRLAWPAIAMLIVFTAIFFGSSKIISKFANGVG----EIKANDFDCKEV 117

APH_1144 67 FGKLAWAAIVMLVVFTAIFFGAGKLIQKFAAGVGSDIIGGNAESFECKGN 116

APH_1132 71 FGRLAWPAIAMLIVFTAIFFGSSKIIGKFAKGIG----ELDADNFDCSKV 116

APH_1133 72 FGRLAWPAIAMLIVFTAIFFGSSKIIGKFANGVG----DLKATEFDCKEV 117

APH_1145 80 FGRSAWFAIAMLIVFSCIFLGGSEFIQKFTEGVG-----DSAGTKHSRVI 124

APH_1136 101 FGRLAWPAIAALVIFTAVFFGAEKVISKFTDGIS----VMQTGNCDTI 144

**. ** .* *..*...* * * **. *. . .

APH_1130 119 AGKNI 123

APH_1131 117 KAG 119

APH_1134 118 AEK 120

APH_1144 117 GATTLSS 123

APH_1132 117 QAEESSSV 124

APH_1133 118 TK 119

APH_1145 125 ASRL 128

APH_1136 145 144
