# Supplementary material for: Whole genome transcription profiling of Anaplasma phagocytophilum in human and tick host cells by tiling array analysis
Source: BMC Genomics. 2008 Jul 31;9:364. doi: 10.1186/1471-2164-9-364 (PMC2527338; doi:10.1186/1471-2164-9-364)
Supplement: Additional file 8 — Examples of other Anaplasmataceae bacteria with multiple virB2 loci. Examples of Anaplasmataceae bacteria with multiple virB2 loci. [file 1471-2164-9-364-S8.doc]

| ***Anaplamatacea* bacteria** | ***virB2* Loci** |  |  |  |  |  |  |  |  |
| --- | --- | --- | --- | --- | --- | --- | --- | --- | --- |
| *Anaplasma marginale* | *AM030, AM044, AM065, AM077, AM082, AM210, AM717, AM723, AM989, AM1061,*  *AM1149, AM1253 , AM1054 , AM1056* | | | | | | | | |
| *Ehrlichia canis* | *Ecaj_0840, Ecaj_0841, Ecaj_0842* | |  |  |  |  |  |  |  |
| *Ehrlichia chaffeensis* | *ECH_1047, ECH_1042, ECH_1043, ECH_1044* | | |  |  |  |  |  |  |
| *Ehrlichia ruminantium* strain Gardel | *ERGA_CDS_08370, ERGA_CDS_08360, ERGA_CDS_08380, ERGA_CDS_08390* | | | | | |  |  |  |
| *Ehrlichia ruminantium* strain Welgevonden | *Erum8000, Erum7990, Erum8010, Erum8020* | | |  |  |  |  |  |  |
| *Wolbachia* endosymbiont of *Drosophila willistoni* | *Wendoof_01000614, Wendoof_01000591, Wendoof_01000615* | | | | |  |  |  |  |
| *Wolbachia* endosymbiont of *Drosophila melagnogaster* | *WD_0351, WD_0651, WD_01289* | |  |  |  |  |  |  |  |
| *Wolbachia* endosymbiont of *Drosophila ananassae* | *WwAna0311, WwAna1590, WwAna1405, WwAna1591* | | | |  |  |  |  |  |

Additional file 8
