# Supplementary material for: Whole genome transcription profiling of Anaplasma phagocytophilum in human and tick host cells by tiling array analysis
Source: BMC Genomics. 2008 Jul 31;9:364. doi: 10.1186/1471-2164-9-364 (PMC2527338; doi:10.1186/1471-2164-9-364)
Supplement: Additional file 1 — Materials and instructions for graphing transcript level data in Artemis [file 1471-2164-9-364-S1.zip › Data for Artemis/Instructions for viewing data.doc]

The authors have prepared data files that allow graphical browsing of the transcription data as described in this publication. This is accomplished with the **free** genome annotation program named “Artemis”.

This free program will run on Mac OSX, Windows, and UNIX computer platforms.

**To view the data in Artemis:**

Download the version of Artemis for your computer from:

[**http://www.sanger.ac.uk/Software/Artemis/**](http://www.sanger.ac.uk/Software/Artemis/)

(There are also complete Artemis manuals at this site)

Place the data files in the folder “Data for Artemis” in the same folder or subdirectory as the Artemis program. This will make navigation to these files a little easier in the following steps.

Start Artemis and click OK on the dialogue.

Go under menu item "File" and select Open and select the file “NC_007797.gbk” from the dialogue box. This is the annotation for the Ap genome. The Aretmis window will open with the annotation loaded.

You now need to load the transcription data as a user plot:

Go under the menu item "Graph" and select “Add User Plot..."

A dialogue box will open, allowing you to select an intensity file for one of the two DNA strands of Anaplasma. Select “Hz plus strand.txt”. and click OK

There will be a pause of 5-30 seconds as your computer imports this data.

Now open the data file for the other strand:

Go under the menu item "Graph" and select “Add User Plot..."

Select “Hz minus strand.txt”.

The window will now look something like this:

Key: HL60 (red line on the plot), HMEC-1 (green line), and ISE6 (blue line)

For more options right click or on a Macintosh hold down the control key while depressing the mouse button.

For even more information see the Artemis manuals and help files at:

[**http://www.sanger.ac.uk/Software/Artemis/**](http://www.sanger.ac.uk/Software/Artemis/)

**File descriptions:**

NC_007797.gbk This is the Anaplasma genome annotation file obtained from:

<ftp://ftp.ncbi.nih.gov/genomes/Bacteria/Anaplasma_phagocytophilum_HZ>

It has been altered by removal of a ribosomal slippage qualifier that generated an error message when the file is imported into Artemis

The files listed below are text files that contain the average raw intensity values (from three array chips each) for the Hz Anaplasma phagocytophilum strain which was grown in HL-60 (red line on the plot), HMEC-1 (green line), and ISE6 (blue line). There is one file for each of the two complementary DNA strands (“plus” and “minus”) These files have been truncated at an intensity of 10,000 to make viewing more predictable. Without this truncation Artemis continuously rescales the data making comparisons difficult.

Hz plus strand.txt

Hz minus strand.txt

**If you have problems or questions please contact us via the email addresses associated with this publication.**
